# Mechanisms and Impact of Rhizosphere Microbial Metabolites on Crop Health, Traits, Functional Components: A Comprehensive Review

**DOI:** 10.3390/molecules29245922

**Published:** 2024-12-15

**Authors:** Qingxia Chen, Yingjie Song, Yuxing An, Yinglin Lu, Guohua Zhong

**Affiliations:** 1 Institute of Nanfan and Seed Industry, Guangdong Academy of Sciences, Guangzhou 510650, China; 2 College of Plant Protection, South China Agricultural University, Guangzhou 510642, China

**Keywords:** rhizosphere microbial metabolites, crop health, plant–microbe interactions, biofertilizers, phytohormones, abiotic stress alleviation

## Abstract

Current agricultural practices face numerous challenges, including declining soil fertility and heavy reliance on chemical inputs. Rhizosphere microbial metabolites have emerged as promising agents for enhancing crop health and yield in a sustainable manner. These metabolites, including phytohormones, antibiotics, and volatile organic compounds, play critical roles in promoting plant growth, boosting resistance to pathogens, and improving resilience to environmental stresses. This review comprehensively outlines the mechanisms through which rhizosphere microbial metabolites influence crop health, traits, functional components, and yield. It also discusses the potential applications of microbial secondary metabolites in biofertilizers and highlights the challenges associated with their production and practical use. Measures to overcome these challenges are proposed, alongside an exploration of the future development of the functional fertilizer industry. The findings presented here provide a scientific basis for utilizing rhizosphere microbial metabolites to enhance agricultural sustainability, offering new strategies for future crop management. Integrating these microbial strategies could lead to increased crop productivity, improved quality, and reduced dependence on synthetic chemical inputs, thereby supporting a more environmentally friendly and resilient agricultural system.

## 1. Introduction

The rhizosphere, a biologically active zone surrounding plant roots, is a hotspot for interactions between plant roots, soil, and a diverse community of microorganisms [1]. These interactions are critical to the functioning of ecosystems and, more specifically, to agricultural productivity [2,3]. The rhizosphere supports an intricate network of microorganisms, including bacteria, fungi, and actinomycetes, that play essential roles in enhancing nutrient availability, improving plant resistance to stress, and contributing to overall soil health [4] (Table 1). The metabolites produced by these rhizosphere microorganisms are increasingly being recognized for their potential to shape plant growth, development, and resilience [5,6]. These metabolites, which include phytohormones, antibiotics, volatile organic compounds (VOCs), siderophores, and exopolysaccharides, each have unique impacts on the plant host and their immediate environment [7,8].

Microbial metabolites are key players in plant health, helping to regulate growth and providing protection against biotic and abiotic stresses [9]. Phytohormones such as indole-3-acetic acid (IAA), gibberellins, cytokinins, and abscisic acid (ABA), produced by various rhizosphere bacteria and fungi, have direct influences on plant physiology, including root elongation, nutrient uptake, and stress response modulation [10,11]. Similarly, antibiotics and VOCs produced by these microorganisms act as natural plant protectants, offering defense against a range of pathogenic organisms and environmental stressors [12,13,14]. For example, metabolites like salicylic acid (SA) and jasmonic acid (JA) are instrumental in activating systemic plant defense mechanisms, enabling plants to better fend off pathogen attacks and adapt to stressful conditions [15,16,17].

The concept of using microbial metabolites as natural enhancers of crop productivity is gaining momentum as agriculture seeks more sustainable alternatives to synthetic chemicals. Traditional farming relies heavily on chemical fertilizers and pesticides, which pose significant environmental challenges, including soil degradation, water pollution, and loss of biodiversity. The use of beneficial rhizosphere microorganisms and their metabolites offers a promising solution by reducing dependence on these chemicals while maintaining or enhancing agricultural productivity [18,19,20]. By leveraging microbial interactions, it is possible to promote plant growth naturally, improve nutrient efficiency, and enhance plant resilience—all of which are essential in the context of global climate change and increasing population pressures [21,22].

Rhizosphere microorganisms produce a variety of secondary metabolites, which were shown to influence plant health in multiple ways [23,24,25]. They assist in nutrient solubilization, such as phosphorus and iron, which are otherwise unavailable to plants in many soil conditions. Siderophores, for example, are iron-chelating compounds secreted by rhizosphere bacteria that enhance the availability of iron—a nutrient that is often limiting in soils [26]. Similarly, microbial exopolysaccharides improve soil aggregation and water retention, thereby indirectly benefiting plant growth, particularly under drought stress conditions [27,28]. These multifaceted roles make rhizosphere microorganisms invaluable partners in agriculture, contributing to soil health, improved crop traits, and ultimately higher yields.

Understanding the mechanisms by which these microbial metabolites influence crop health, traits, functional components, and yield is crucial for developing innovative strategies aimed at enhancing agricultural sustainability. Advances in research have demonstrated that microbial metabolites can be integrated into crop management practices as biostimulants and biofertilizers, contributing not only to higher yields but also to improved nutritional quality of crops [29,30]. For instance, microbial inoculants that produce growth-promoting hormones and stress-alleviating compounds can help crops cope with adverse conditions, such as drought and soil salinity, which are increasingly common due to climate change [31].

This comprehensive review aims to outline the various mechanisms through which rhizosphere microbial metabolites impact plant health and yield, as well as to discuss their potential applications in sustainable agriculture. Additionally, it identifies the challenges associated with their practical use and suggests strategies to overcome these obstacles to ensure effective field applications. By integrating microbial solutions into agricultural practices, the reliance on chemical inputs can be reduced, leading to more sustainable, environmentally friendly, and resilient cropping systems. The findings presented in this review provide a scientific basis for developing new functional biofertilizers and highlight the significant potential of rhizosphere microbial communities in transforming modern agriculture.

**Table 1:** Sources and functions of common microbial metabolites.

Classification	Metabolite Source	Secondary Metabolites	Secondary Metabolite Effects	References
Bacteria	*Bacillus* genus, *Bacillus* subtilis, *Bacillus* licheniformis, *Bacillus* amyloliquefaciens, *Bacillus* thuringiensis, *Bacillus* beleriensis	Mycobacterium, Ichthyosporin, Dictyostelium and antimycobacterial bacillin	Inhibition of Colletotrichum, Wilt, Melon Fruit Rot, Tomato Grey Mildew control.	[32,33,34,35,36,37]
	*Pseudomonas* spp. *Pseudomonas* malodorata, *Pseudomonas* globulus, *Pseudomonas* luteus	2,4-Diacetylresorcinol, Nitrotyrosin, Cyanine, Phenazine, Nitropyridin, Pseudomonas syringae, etc.	Inhibition of pathogenic bacteria, leaf blight, wheat total blight control.	[38,39,40,41]
	*Burkholderia* spp.	Xylomycin, gummycotoxin, antimicrobial, pyrolnitrin, pseudane, etc.	Rice blight control.	[42,43,44]
Fungus	*Trichoderma* spp.	Xylomycin, gummycotoxin, antipeptide, pyrolnitrin, etc.	Inhibit wilt and total blight, control root rot of wheat and rice blight.	[45,46,47,48,49,50]
	*Scopulariopsis*	gliovirin	Inhibit Ultimate Rot.	[51,52,53,54,55,56,57]
	*Trichoderma* harzianum	Trichothecene	Inhibition of fungi and viruses, control of wilt disease of cotton seed and other crops.	[58,59,60,61,62,63]
Actinomycetes	*Streptomyces*	Validamycin, avermectin, Abamectin, streptomycin, etc.	Inhibition of plant pathogenic fungi, rice blast, blight, pest control.	[64,65,66,67]

## 2. Impact of Microbial Metabolites on Crops

The impact of rhizosphere microbial metabolites on crop health is a crucial area of research, offering insights into enhancing agricultural productivity and sustainability. Rhizosphere microorganisms directly affect crop growth, development, and health by secreting a diverse range of metabolites, including phytohormones, antibiotics, vitamins, and other bioactive compounds. These metabolites are essential in mediating plant–microbe interactions that influence nutrient uptake, disease resistance, and environmental stress response. The effects of rhizosphere microbial metabolites on crop health can be summarized in three main aspects:

(1) Promoting plant growth: Rhizosphere microorganisms provide plants with effective nutrients by converting organic matter into bioavailable forms and secreting growth-promoting compounds like vitamins and phytohormones. For example, auxins and cytokinins produced by rhizobacteria help in enhancing root development, increasing nutrient uptake efficiency, and ultimately promoting overall plant growth and vigor [68,69,70].

(2) Preventing and controlling diseases: Microorganisms in the rhizosphere can secrete antibiotics and other antimicrobial substances that inhibit the proliferation of pathogenic bacteria and fungi, helping plants resist soil-borne diseases. This biocontrol mechanism reduces the need for chemical pesticides, thereby contributing to sustainable agricultural practices [71,72,73].

(3) Responding to environmental stress: Rhizosphere microorganisms play a key role in helping plants cope with unfavorable environmental conditions, such as high temperatures, drought, and soil salinity. They achieve this by regulating soil nutrient cycling, producing osmoprotectants, and secreting anti-stress compounds, thereby enhancing the plant’s resilience to abiotic stress [74].

The mechanisms by which these microbial metabolites influence crop health include:

(1) Root Exudation and Microbial Stimulation: Plant roots release organic acids, sugars, and secondary metabolites that specifically promote the growth of beneficial microorganisms. These root exudates create a conducive environment for beneficial microbes while simultaneously inhibiting pathogenic organisms through both direct and indirect immune responses [75,76,77].

(2) Microbial–Plant Interactions: The interactions between rhizosphere microorganisms and plant roots are dynamic and mutually adaptive. As the plant grows and environmental conditions change, the composition and function of the microbial community adjust accordingly, thereby maintaining crop health and productivity. This equilibrium allows the plant to optimize its use of available microbial metabolites, ensuring that growth and defense needs are met effectively [78].

### 2.1. Growth Regulating Substances

Microbial metabolites in the rhizosphere are crucial in regulating plant growth and development by producing growth-promoting phytohormones. These metabolites include auxins, gibberellins, cytokinins, abscisic acid, ethylene, salicylates, jasmonates, and other growth regulators (Table 2). These compounds play a vital role in enhancing crop health, increasing productivity, and improving resilience to environmental stress.

#### 2.1.1. Auxins (Indole-3-Acetic Acid, IAA)

Auxins, particularly IAA, are among the most well-studied microbial metabolites with significant effects on plant growth and development. Produced by rhizosphere bacteria such as Pseudomonas, Azospirillum, and Bacillus, IAA modulates root elongation, lateral root formation, and overall root architecture, which enhances nutrient uptake and plant vigor. IAA plays a critical role in root gravitropism and helps plants adapt their root systems to better explore the soil for water and nutrients [107]. Studies have shown that inoculation with IAA-producing bacteria can lead to increased root biomass, improved stress tolerance, and ultimately increased crop yields. Additionally, microbial IAA promotes symbiotic relationships, such as mycorrhizal associations, which further boost nutrient acquisition and enhance plant resilience against stress [108].

#### 2.1.2. Gibberellins

Gibberellins are phytohormones synthesized by both plants and certain rhizosphere microorganisms, such as Rhizobium, Bacillus, and Penicillium species [83,84]. These metabolites play a fundamental role in promoting stem elongation, seed germination, flowering, and fruit development. Gibberellins also contribute to breaking seed dormancy, enabling seeds to germinate even under less-than-ideal conditions. Research has demonstrated that gibberellin-producing microbes significantly enhance plant growth under suboptimal conditions, such as saline and nutrient-poor soils [109]. Moreover, these microorganisms can alleviate the negative impacts of abiotic stress by promoting cell expansion and elongation, thereby allowing plants to maintain their growth rate despite environmental challenges. The application of gibberellin-producing microbes is particularly valuable for crops that experience growth restrictions due to environmental stressors, thereby contributing to increased productivity and yield stability.

#### 2.1.3. Cytokinins (CTK)

Cytokinins, produced by rhizosphere microbes like Arthrobacter, Azotobacter, and Bacillus, are essential for promoting cell division, shoot initiation, and delaying leaf senescence [85,86,87,88,89]. Cytokinins influence many aspects of plant growth, such as stimulating chloroplast development, enhancing nutrient remobilization, and promoting lateral bud formation, which leads to increased biomass production. The application of cytokinin-producing bacteria is associated with delayed leaf aging, improved chlorophyll retention, and enhanced photosynthetic activity, which contribute to prolonged crop productivity [110]. Cytokinins also play an essential role in both biotic and abiotic stress adaptation, such as salinity and drought [111]. They enhance the plant’s ability to adapt to harsh environmental conditions by regulating water balance, improving antioxidant activity, and influencing root-to-shoot signaling [112]. This versatility makes cytokinins a crucial component in optimizing agricultural production, especially under stress-prone environments.

#### 2.1.4. Abscisic Acid (ABA)

ABA is a crucial phytohormone that regulates numerous physiological processes, including stomatal closure, seed dormancy, and stress response. While it is primarily synthesized by plants, several microbial strains, including fungi such as Aspergillus and some bacterial species, were also reported to produce ABA [90,91,92,93]. In the rhizosphere, microbial ABA production contributes significantly to enhancing plant tolerance to abiotic stresses, such as drought, high salinity, and temperature extremes [113]. ABA modulates stomatal closure to reduce transpiration under water-deficit conditions, helping plants conserve water. It also regulates gene expression related to stress tolerance, promoting the synthesis of protective proteins and metabolites. ABA-producing microorganisms have the potential to be used as biostimulants to improve crop resilience under harsh conditions, offering a natural and sustainable alternative to synthetic growth regulators [114,115]. The successful production of microbial ABA under controlled fermentation conditions also opens opportunities for large-scale application in agriculture.

#### 2.1.5. Ethylene (ETH)

Ethylene is a gaseous phytohormone known for its involvement in regulating plant growth, development, and response to environmental stress. It is produced by several rhizosphere microorganisms, including soil bacteria such as *Pseudomonas*, *Bacillus*, *Escherichia coli*, as well as some yeast and fungal species [94]. Ethylene modulates various plant processes, such as seed germination, inhibition of root elongation, root hair development, and fruit ripening. In addition to its role in growth regulation, ethylene is also crucial for plant adaptation to biotic and abiotic stresses [116]. During pathogen attacks or mechanical wounding, ethylene production is increased, triggering defense responses, including the expression of defense-related proteins and metabolites [117]. Microbial ethylene also influences root hair proliferation, which increases the root surface area, thereby enhancing water and nutrient absorption [118]. In the context of agricultural production, the manipulation of ethylene production by microbial inoculants can help regulate plant developmental processes, ensuring better growth and stress tolerance. Ethylene also plays an essential role in coordinating with other phytohormones, such as auxins and jasmonates, to fine-tune plant responses, making it a versatile target for improving crop resilience and productivity [119].

#### 2.1.6. Salicylate (SA)

SA is an emerging phytohormone favored by many researchers and widely recognized for its role in biotic and abiotic stresses in plants [120]. SA is an active secondary metabolite found in bacteria, fungi and plants. It is extensively involved in many physiological phenomena in plant growth and development and is actively involved in the response to biotic and abiotic stresses, thus conferring broad-spectrum resistance [121,122]. SA is considered to be an important phytohormone that regulates all aspects of plant growth, environmental stresses, and defense responses to defense responses to pathogens [123]. In addition to plants, a large number of bacterial species, such as *Pseudomonas*, *Bacillus*, *Azoospirillum*, *Salmonella*, *Vibrio*, *Yersinia*, and *Mycobacterium*, have been reported to synthesize salicylates via the NRPS/PKS biosynthetic gene cluster. This bacterial salicylate production is usually associated with the biosynthesis of small ferrous ion chelating molecules, salicylidene-derived iron carriers called catecholates, under iron-restricted conditions [124]. Salicin is a notable phenolic glycoside derived from plants including *Salix* and *Populus* genus and has multiple biological activities such as anti-inflammatory and antiarthritic, anticancer, and antiaging effects [95]. Some bacteria and a few fungi can produce salicylate hydroxylase to degrade SA to suppress plant defense and increase their virulence [123]. In addition, SA is an important platform chemical widely used in the cosmetic and pharmaceutical industries [125].

#### 2.1.7. Jasmonate (JA)

JA is a fatty acid derivative that controls several plant processes including growth, development and defense [126]. JA is a plant hormone with essential roles in reproductive development and in the regulation of plant responses to multiple stresses. Studies have found that inoculation with the rhizobia induces the JA pathway in *Medicago truncatula*, and blocking the JA pathway significantly reduces the number of infection threads [127]. Exotic pathogens and other forms of biotic stress infections can stimulate the rapid synthesis and accumulation of JA and its derivatives, thereby promoting the expression of defense-related genes and the production of defense-related metabolites [128]. Examples include the diterpenoid plant antitoxin momilactones and the flavonoid plant antitoxin sakurin [127,129,130]. Several studies confirmed this claim and found that JA induced the accumulation of a range of terpenoids that inhibited the spread of Xanthomonas oryzae pv. oryzae, thereby creating resistance to bacterial leaf blight (BLB) in rice [131,132,133]. It was also shown that jasmonic acid treatment enhances cold tolerance in peach fruits by modulating ethylene and sugar metabolism [134].

#### 2.1.8. Others

Pironetin is produced by *Streptomyces*, a metabolite with plant-growth-regulating activity, and is a pyran structural ketone structure. Pironetin does not inhibit the synthesis of gibberellins, but has the effect of inhibiting the heightening of the crop plant, and after the addition of gibberellins, the plant can resume growth [135]. It does not affect the biochemical synthesis of amino acids in plants, but it can increase the amount of ethylene produced by plants [136]. Bilonisin is moderately toxic to humans and animals, but it is highly toxic to aquatic organisms. Oxamycin, also known as cyclo-serine (cyoloserin), can be produced by a variety of *streptomycetes* and can be used in agriculture to regulate plant growth and increase the sugar content of plantain [137]. It is also effective against Gram-positive and Gram-negative bacteria and can be used as an antimicrobial agent, which is safe for humans and animals. These microbial growth regulators collectively enhance plant growth, improve stress resilience, and reduce the need for synthetic inputs in agriculture, contributing to more sustainable and productive cropping systems [138,139,140].

### 2.2. Siderophores

Siderophores are iron-chelating compounds secreted by various soil microbes, including Pseudomonas, Bacillus species and Rhizobium [141]. These metabolites enhance the availability of iron to plants, which is often a limiting nutrient in soils. Siderophore-producing microbes were shown to alleviate iron deficiency in crops, leading to improved plant growth and higher yields.

Plant-growth-promoting bacteria (PGPB) are able to colonize the inter-root and promote iron uptake by plants [142]. Iron carriers can be released under appropriate conditions, thereby increasing and regulating iron bioavailability. These microorganisms are capable of producing ferric ions under iron-deficient conditions. Iron ions are key components of various metabolic pathways within the cell. Iron is essential for many plant processes, including photosynthesis. Iron ions are also required for chlorophyll synthesis and the general functioning of photosynthetic bodies. In addition, a number of other proteins and protein complexes involved in electron transfer and mitochondrial oxidative phosphorylation during chloroplast photosynthesis depend on iron: non-heme Fe-S proteins (e.g., ferredoxin), heme proteins (e.g., catalase and peroxidase), and cytochromes. In addition, iron acts as a cofactor in the synthesis of many plant hormones, such as ethylene and 1-aminocyclopropane-1-carboxylate. Iron-producing carrier PGPBs promote plant growth and improve host plant nutrition, but PGPBs have other benefits for plants. For example, they can solubilize phosphate and fix atmospheric nitrogen [70]. Considering that the synthesis of iron carriers requires mineral phosphates and mineral (organic) nitrogen, this association of PGPBs is considered a potential microfertilizer. Other PGPB may reduce the impact of soil plant pathogens by producing antimicrobial compounds and extracellular enzymes that affect crop growth. Iron carrier-producing microorganisms produce large amounts of iron-chelating compounds, thereby accelerating physiological and biochemical processes in plants under unfavorable conditions. Based on their chemical properties, iron carriers can be categorized as catecholates, isohydroxamates, carboxylates and mixed iron carriers [143].

#### 2.2.1. Catecholate Iron Carriers

In catecholate type iron carriers, iron(III) ions are bound to hydroxyl or catecholate groups. Chelation with iron(III) results in the formation of a six-coordinated octahedral complex with two oxygen atoms involved in each catecholate group. All catecholic acid-based iron carriers are derivatives of salicylic acid or 2,3-dihydroxybenzoic acid (2,3-DHBA). Catecholate-type iron carriers, such as spirochetes, are produced by Spirochetes brasiliensis nitrogen-fixing bacteria in iron-deficient media. Lipid-bearing nitrogen-fixing spirochetes produce 2,3-DHBA and 3,5-DHBA coupled to threonine and lysine, which also have iron carrier activity. Catecholates, e.g., enterobactin found in E. coli strains, have been described as having the highest iron affinity [144].

#### 2.2.2. Iron Isohydroxamic Acid Carriers

Iron isohydroxamic acid carriers have the structure C(=O)N-(OH)R, where R is an amino acid containing two oxygen atoms or a derivative thereof, and form a bis-coordinating ligand with iron ions. Each of the iron carriers can form hexa-coordinating ligands and octahedral complexes with iron(III) ions. When an isohydroxamic acid salt binds to iron(III), its functional group loses a proton from the hydroxylamino group (-NOH) to form a bis-coordinating ligand [145]. Bacillus megaterium ATCC 19213 is known to produce two types of iron isohydroxamic acid carriers (shizokinen and N-deoxyshizokinen) under iron-limiting conditions [146]. In addition to having a high affinity for iron(III) ions, these iron carriers are also capable of chelating aluminum [146]. The pea rhizobium IARI 917 is also known to produce schizokinen iron carriers [147]. Pantoea vagans C9-1 produces isohydroxamic acid-type desferrioxamine-like iron carriers [145]. Certain strains of Rhizobium radiodurans are capable of producing isohydroxamic acid type iron carriers, for example, Rhizobium chinense produces an iron carrier called rhizokinin [148]. Rhizobium chinense 1021 produces a variant of rhizobactin called rhizobactin 1021.Vicibactin is a cyclic tri-isohydroxamic acid iron carrier found in Rhizobium pea and Rhizobium cauliflower [148].

#### 2.2.3. Carboxylate and Hybrid Iron Carriers

Carboxylate iron carriers bind iron through carboxyl and hydroxyl groups. Carboxylate iron carriers in PGPB have not been described in the literature. However, these iron carriers are present in hybrid iron carriers. In addition to the above types, some iron carriers contain multiple iron chelating groups and are, therefore, categorized as hybrid iron carriers. Notably, iron carriers exhibit antifungal properties. One of the most studied iron carriers with direct antifungal properties is the pusillanimals produced by Pseudomonas aeruginosa, which inhibit pathogen development by increasing competition for iron: fungal iron carriers typically have a lower affinity for iron(III) than bacterial iron carriers [145]. For example, it was shown that Pseudomonas fluorescens WCS374r (Psb374) is required for the induction of iron carrier-mediated resistance in rice infected with rice giant zoochlorella. Inoculation of soil with the mutant strain Psb374 deficient in Pseudomonas putida and infection of rice leaves with rice giant zoochlorella 4–5 days later showed that the iron-carrier mutant bacteria did not inhibit disease in rice compared to wild-type Pseudomonas fluorescens [149]. Further future analysis of the biochemical, molecular biological and physiological characterization of bacterial synthesis of iron carriers and their utilization by plants would enable the creation of effective microbial agents to improve soil fertility and increase plant biomass, which is highly relevant to sustainable agriculture [145].

In addition to iron, iron carriers can chelate biogenic metals such as copper, cobalt, and nickel, as well as toxic metals such as aluminum, gallium, and lead, and radionuclides such as uranium, albeit with a lower affinity. This property has brought iron carriers to the forefront of interest in fields such as agriculture, bioremediation, medicine, and cancer research [150]. Inoculation of SPB isolates can promote plant selenium uptake by modulating native bacterial taxa. These results demonstrate that SPB can promote plant selenium uptake through multiple pathways and can be used for selenium biofortification in subtropical soil crops [151].

Studies have shown that when intercropping faba bean, it can resist the enrichment of pathogenic bacteria by increasing bacterial diversity, changing the structure of bacterial communities, and enhancing the complexity of bacterial networks, while corn-enriched iron-producing carrier bacteria and specific antagonistic bacteria can also work together to resist the growth and development of pathogenic fungi, which ultimately protects the faba bean plant from invasion of pathogenic fungi and improves crop yields [152]; Pseudomonas aeruginosa 1502IPR-01 improves iron nutrition and yield of peanuts by secreting iron-loaded iron pyoverdine. Specific Pseudomonas spp. obtained from maize inter-roots by cross-enrichment secreted the iron-carrier pyoverdine to increase inter-root iron effectiveness, which in turn improved peanut iron nutrition [153]. Strains of Pseudomonas spp. from the genus Pseudomonas fluorescens produce 2,4-diacetylphloroglucino l (DAPG), which can help plants to resist pathogenic fungal infections. In addition, pyoverdine can affect the interaction between tomato and pathogenic Penicillium spp. It was shown that the combined function of the antimicrobial agent DAPG and the iron carrier pyoverdine as an exo-metabolite explains most of the inhibitory activity of the strongly antagonistic strain Pseudomonas brassicacearum R401 [154].

### 2.3. Lipopeptides

Lipopeptides are a class of microbial metabolites with strong antimicrobial properties, produced by species such as Bacillus subtilis. These compounds inhibit soil-borne pathogens, thereby protecting crops from disease. The use of lipopeptide-producing biocontrol agents is an emerging strategy in integrated pest management (IPM), helping to make crops healthier and less dependent on chemical pesticides. Several inter-root microorganisms produce antibiotics such as DAPG from Pseudomonas fluorescens. These compounds inhibit plant pathogens and promote plant health. Antibiotic-producing microorganisms promote crop resistance to disease and are increasingly being incorporated into sustainable agricultural practices [155]. Surfactin, iturin, fengycin, bacillomycin, and polymyxins are the most widely studied lipopeptides in Bacillus. The different types of lipopeptides produced by bacteria and their effects on plant pathogens are summarized below (Table 3).

### 2.4. Antibiotics

Several rhizosphere microbes produce antibiotics, such as DAPG from Pseudomonas fluorescens. These compounds suppress phytopathogens, enhancing plant health. Antibiotic-producing microbes promote crop resistance to diseases and are increasingly integrated into sustainable agricultural practices [175]. Agricultural antibiotics of microbial origin have become the main varieties of biopesticides because of their high efficiency, green and environmental protection characteristics, etc. It is of great significance to actively screen and develop new microbial-origin agricultural antibiotics to promote the development of biopesticides and agricultural production in China. The following is a brief summary of the new microbial-derived agro-antibiotics developed in the past 10 years (Table 4).

### 2.5. Volatile Organic Compounds (VOCs)

Microbial VOCs, including compounds like acetoin and 2,3-butanediol, are recognized for their ability to induce plant growth and defense mechanisms [216]. Studies have shown that VOCs produced by rhizobacteria can enhance root growth, improve nutrient uptake, and trigger systemic resistance against pathogens, making them promising candidates for crop enhancement [217,218]. In addition, microbial sources of VOCs have the advantages of being effective, safe, environmentally friendly, easily degradable and residue-free, which are increasingly emphasized and favored by researchers in various countries [219,220]. Many studies now show that VOCs produced during the growth and reproduction of some antagonistic microorganisms have an extremely strong bacteriostatic effect and can act synergistically to inhibit or even kill post-harvest pathogenic bacteria of fruits and vegetables, and some of them can also promote plant growth and crop yield [221]. Many bacteria are capable of producing VOCs, which consist mainly of alcohols, ketones, terpenes and other carbon-containing compounds. For example, Rhizobium spp. produce certain compounds that promote plant growth. Fungi, especially molds, also produce a variety of VOCs, and these compounds are commonly used in the processing of food and pharmaceuticals, as well as in the decomposition of organic matter (Table 5). For example, Aspergillus produces some compounds that are used in the production of biofuels.

In agricultural production, VOCs produced by certain microorganisms are used in biocontrol, such as against plant pathogens (Table 5). For example, Pseudomonas fluorescens produces specific VOCs that inhibit the development of plant diseases.

### 2.6. Exopolysaccharides (EPS)

Exopolysaccharides (EPS), secreted by rhizobacteria, are complex polysaccharides that play a crucial role in soil aggregation, enhancing soil structure, water retention, and overall soil fertility [252]. EPS-producing microorganisms contribute significantly to the physical and biological properties of the soil, thereby improving the growth conditions for plants. These microbial EPS serve as natural soil conditioners, binding soil particles together to form aggregates that enhance soil porosity and stability. By improving these properties, EPS help facilitate root penetration, increase water availability, and enhance nutrient uptake, ultimately promoting healthier plant growth and increased crop resilience [253].

EPS are water-soluble polysaccharides secreted by inter-root bacteria during their growth and metabolism. They enhance soil aggregation by binding soil particles into larger, more stable aggregates (>0.25 mm), which contributes significantly to soil structure improvement. Soil aggregates are fundamental units of soil structure, and their number, size distribution, and stability determine soil fertility, water retention, and resistance to erosion [254]. Increased soil stability due to EPS production leads to improved root infiltration, enhanced water retention, and increased nutrient utilization efficiency, providing critical benefits under conditions of abiotic stress, such as drought [255].

Research has demonstrated that EPS-producing microorganisms, such as Bacillus subtilis (Accession No. MT742976) and Azospirillum brasilense (Accession No. MT742977), produce high levels of EPS that help mitigate drought stress in crops like wheat [255]. These beneficial microbes not only improve soil aggregation but also enhance the soil’s physical properties, including porosity and permeability, which are essential for effective root growth and nutrient uptake. By facilitating the formation of water-stable macroaggregates, EPS indirectly enhance the availability of water to plants, ensuring that roots can absorb water even under limited moisture conditions. This ability is especially valuable for maintaining productivity in arid and semi-arid regions [256].

EPS also play a significant role in enhancing carbon sequestration by stabilizing organic carbon within soil aggregates, thereby contributing to long-term carbon storage [257]. The increased soil organic matter content further enhances soil fertility and promotes beneficial microbial activity in the root zone. Moreover, EPS provide a favorable microenvironment for beneficial microbial communities, which in turn interact with plant roots to promote plant growth and stress tolerance [258].

Several studies have highlighted the positive effects of EPS-producing bacteria on soil and plant health. For instance, seed treatment with EPS-producing bacteria was shown to increase the proportion of water-stable soil aggregates, promote the growth of tomato seedlings, and reduce damage caused by greening blight. Soil inoculation with high-EPS-producing strains like Bacillus amyloliquefaciens (HYD-B17), Bacillus licheniformis (HYTAPB18), and Bacillus subtilis (RMPB44) resulted in enhanced soil cementation, significantly increasing the number of water-stable macroaggregates and thus improving the physical stability of the soil [259]. The positive effect on soil structure helps maintain a favorable root environment, further facilitating the uptake of water and nutrients.

EPS-producing bacteria also have a profound effect on crop resilience to salt stress. Salt-tolerant strains such as Stenotrophomonas and Bacillus species were found to enhance plant growth under high salinity conditions by improving soil structure and alleviating salinity stress [260,261]. For example, Bacillus subtilis GBW HF-98 improves soil aggregation in saline and alkaline conditions by producing EPS that mitigate adverse soil properties. This, in turn, was shown to promote the growth of tomatoes by reducing soil salinity effects, which are often detrimental to plant growth. In addition to improving soil aggregation and promoting plant growth, EPS-producing bacteria contribute to overall soil health by enhancing the microbial community structure and facilitating interactions between plants and beneficial microbes [262]. The polysaccharides produced by these microorganisms also act as signaling molecules, facilitating communication between plant roots and rhizosphere microbes, thereby promoting the establishment of beneficial microbial associations such as mycorrhizae and nitrogen-fixing bacteria. These interactions further enhance nutrient availability and contribute to increased plant biomass and yield [256].

Despite the numerous benefits offered by EPS-producing bacteria, the strain resources currently applied to soil improvement are still limited. The production of EPS is highly sensitive to environmental factors such as temperature, soil pH, and salinity, which can limit their effectiveness in the field. Therefore, it is essential to screen for robust microbial strains that not only produce high levels of EPS but also exhibit traits such as disease resistance, salinity tolerance, and environmental stability. The development of such strains could provide valuable candidates for composite functional microbial inoculants, enhancing the efficacy of biological soil conditioners and enabling their large-scale industrial application in sustainable agriculture [263].

### 2.7. Terpenoids and Polyketides

Terpenoids and polyketides are secondary metabolites with potent antimicrobial properties, produced by various rhizosphere bacteria and fungi. For instance, the production of 2-methylisoborneol by Streptomyces species and various polyketides by Pseudomonas strains were shown to inhibit the growth of a wide range of plant pathogens [264,265]. These metabolites play a significant role in natural plant disease resistance, providing a sustainable alternative to chemical fungicides [266].

Terpene-producing microorganisms are capable of producing various types of terpene compounds, which include organic compounds such as aromatic hydrocarbons, aliphatic compounds and alkaloids. Some specific bacteria, such as Streptomyces and Fungi, are representative of this group of microorganisms. For example, certain species of the genus Streptomyces are capable of producing broad-spectrum antibiotics such as erythromycin, while certain species of the genus Penicillium are capable of producing anticancer substances. As terpenoids are one of the most widely distributed and diverse natural products in nature, and have a wide range of applications and an increasingly strong market demand, artemisinin [267], for example, Qinghaosu is a terpenoid [268]. Terpenoids are widely used in the pharmaceutical industry due to their pharmacological activities such as anti-inflammatory, antioxidant, and inhibition of tumor cell proliferation. In recent years, the synthesis of terpenoids using microorganisms has received widespread attention.

Polyketides are long-chain molecules consisting of a series of carbon chains and are widely used in the production of antibiotics, antitumor drugs, and agrochemicals [269]. Such microorganisms usually have complex biosynthetic pathways that are capable of producing polyketide compounds containing multiple ring structures. Polyketides are a widespread class of microbial secondary metabolites and an important source of natural medicines, and their structural diversity has led to a wealth of biological activities [270]. For example, erythromycin, which has a macrolide structure found in Saccharopolyspora erythraea, is a widely used first-line drug for the treatment of bacterial infections [271]; amphotericin B, which has a polyene skeleton, is produced by Actinomadura produced for the treatment of fungal infections; the anthracycline drug zorubicin (daunorubicin) is used in tumor therapy; and avermectin, derived from Streptomyces avermitilis, is a hexadecanoid macrolide compound with insecticidal and acaricidal activity. Mixed terpenoids are natural products of hybridization of terpenoids and polyketides, which are widely found in plants, fungi and bacteria, and have good biological activities. Penicillium sp. KM18029 is an endophytic fungus isolated from Aconitum brachypodum Diels, which was found to be able to synthesize mixed terpenoids [272,273]. The inhibitor of histone deacetylase, avermectin, is a hexadecanoid macrolide compound with insecticidal activity. The histone deacetylase inhibitor suberoylanilide hydroxamic acid (SAHA) is a chemical epigenetic modifier that activates clusters of silent genes in microorganisms and is commonly used for microbial novel secondary metabolite mining [274].

Terpenoids and polyketides of microorganisms have a wide range of potential applications in areas such as drug discovery, agrochemical production, and biotechnology. For example, some terpenoids have unique biological activities that can be used in the discovery and development of new drugs. Meanwhile, polyketides are widely used in the fields of antimicrobial, antitumor and plant growth regulators due to their wide range of biological activities and good stability [269].

### 2.8. Role of Microbial Enzymes in Crop Health and Nutrient Assimilation

Microbial enzymes play a critical role in enhancing crop health by facilitating the breakdown of organic matter, which increases the flow of nutrients in the soil and their subsequent assimilation by plants [275]. These enzymes, produced by soil microorganisms such as bacteria and fungi, include cellulases, amylases, proteases, and phosphatases [276]. By breaking down complex organic materials into simpler, bioavailable forms, microbial enzymes contribute to improving soil fertility and nutrient cycling.

Enzyme production by beneficial microbes supports plant growth by releasing nutrients such as nitrogen, phosphorus, and potassium, which are essential for plant development [277]. For instance, phosphatases produced by certain bacterial species solubilize organic phosphorus compounds, converting them into inorganic phosphate that can be readily absorbed by plants. Similarly, cellulases and other enzymes help decompose plant residues, releasing carbon and other nutrients back into the soil. The agricultural enzyme market is rapidly growing due to the increased focus on sustainable farming practices. Enzymatic degradation of organic matter helps improve nutrient availability, reduce the need for chemical fertilizers, and enhance soil health, making enzymes a promising tool for sustainable crop management [278]. Additionally, microbial enzymes are instrumental in promoting plant resilience to environmental stresses by improving nutrient acquisition and supporting overall plant vigor [279].

Integrating microbial enzyme production into biofertilizer formulations offers a significant advantage in promoting agricultural sustainability [280]. By enhancing nutrient uptake and reducing dependence on synthetic fertilizers, microbial enzymes support a more environmentally friendly approach to crop production. Therefore, incorporating microbial enzymes as part of an integrated soil fertility strategy is crucial for enhancing agricultural productivity in an eco-friendly manner.

## 3. Mechanisms of Microbial Metabolites Influencing Crop Health

### 3.1. Induction of Plant Defense Mechanisms

Microbial metabolites such as jasmonic acid (JA), salicylic acid (SA), and ethylene (ET) are well-known for their roles in activating plant defense pathways [281]. These metabolites are involved in systemic acquired resistance (SAR) and induced systemic resistance (ISR), which provide crops with enhanced protection against various pathogens, pests, and environmental stresses. Beneficial rhizosphere microorganisms, including bacteria and fungi, stimulate crop growth and induce immunity through complex hormone signaling pathways, such as SA, JA, ET, and other plant defense-related compounds [282].

ISR is a mechanism triggered by certain plant-beneficial rhizobacteria and fungi that modulates plant hormone signaling [283]. These beneficial microbes regulate gene expression involved in ISR, which leads to the synthesis of secondary metabolites, defense enzymes, and volatile compounds, thereby inducing systemic plant defense mechanisms [284]. This process enhances plant resilience against a wide variety of biotic stresses, including pathogens, insects, and parasitic organisms [285].

In response to microbial metabolites, plants activate different defense responses, including the production of reactive oxygen species (ROS), accumulation of phytoalexins, and synthesis of pathogenesis-related (PR) proteins [286]. For instance, SA plays a crucial role in SAR by triggering the accumulation of PR proteins, which enhance the plant’s immune system and provide resistance to pathogens across the entire plant. Similarly, JA and ET pathways activate ISR, which primes plants to respond more effectively to pathogen attacks by accumulating defense metabolites [287].

Plants possess sophisticated defense mechanisms to protect themselves, including SAR, ISR, systemic wound response, and systemic gene silencing [288]. While some beneficial rhizobacteria activate the SA-dependent SAR pathway through SA production on the root surface, others stimulate different signaling pathways independent of SA, such as the JA and ET-dependent ISR pathways [289,290]. These multiple signaling pathways interact synergistically, providing robust protection against a wide spectrum of biotic stresses, reducing reliance on chemical pesticides, and fostering more sustainable crop management practices [281].

### 3.2. Alleviation of Abiotic Stress

Rhizosphere microorganisms play an essential role in helping plants cope with abiotic stresses, such as drought, salinity, heavy metal toxicity, and extreme temperatures. These beneficial microbes produce a variety of metabolites, including osmoprotectants, antioxidants, and metal chelators, that mitigate stress impacts and enhance plant tolerance. Osmoprotectants, such as proline, glycine betaine, and trehalose, help maintain osmotic balance within plant cells, while antioxidants such as catalase, peroxidase, and superoxide dismutase alleviate oxidative damage caused by environmental stress [291].

Salicylic acid (SA) is a well-documented microbial metabolite that plays an important role in enhancing plant tolerance to various abiotic stresses, including high temperatures, drought, and salinity [121,122]. SA modulates stress-responsive gene expression and enhances the synthesis of protective metabolites, thereby helping plants adapt to unfavorable conditions. By boosting antioxidant enzyme activity, SA reduces oxidative stress and enhances plant vigor under challenging environmental conditions.

Arbuscular mycorrhizal fungi (AMF), particularly those belonging to the Glomeromycota phylum, are also known for their role in alleviating abiotic stress in plants [292]. AMF colonize plant roots and form symbiotic associations that enhance drought tolerance by improving water uptake and increasing antioxidant enzyme activities [293]. These fungi help reduce oxidative stress, improve water-use efficiency, and increase plant biomass under water-limited conditions. Similarly, beneficial rhizosphere microorganisms such as Streptomyces were found to enrich the soil environment during drought, producing bioactive metabolites that alleviate drought-induced stress and promote plant growth [294].

Plants under abiotic stress also recruit beneficial microbial communities by increasing the release of root exudates, including primary metabolites such as amino acids, fatty acids, and lysophosphatidylcholine [295]. These exudates serve as signaling molecules, attracting beneficial microbes capable of mitigating the stress [296]. For example, when plants are exposed to low doses of pesticides, microbial communities can provide compensatory effects that reduce the phytotoxic impact, thereby promoting plant growth. Microbes such as Nocardia, Chlamydomonas, and Sphingomonas are capable of degrading pesticides and minimizing their negative effects on plant health [297].

Salt stress is another major abiotic factor that affects crop productivity, particularly in arid and semi-arid regions. Beneficial rhizosphere microorganisms, such as salt-tolerant strains of Stenotrophomonas and Bacillus, produce EPS and other metabolites that alleviate the impact of salt stress by improving soil structure and enhancing soil aggregation [260,261]. For example, Bacillus subtilis GBW HF-98 enhances soil aggregation through EPS production, thereby reducing salinity stress and improving plant growth under saline conditions.

Overall, rhizosphere microbial metabolites are key players in supporting plant health under both biotic and abiotic stresses. These beneficial metabolites provide plants with mechanisms to overcome challenges, ensuring growth and productivity even in adverse environmental conditions. By harnessing these microbial interactions, agricultural systems can be made more resilient and less reliant on chemical inputs, promoting sustainability and improving overall crop productivity. The use of microbial inoculants and bioformulations based on these beneficial mechanisms offers promising opportunities for enhancing agricultural sustainability, increasing yields, and contributing to food security in the face of global climate change and other environmental challenges [298].

## 4. Influence of Microbial Metabolites on Crop Traits

### 4.1. Modulation of Plant Growth and Development

Rhizosphere microbial metabolites like auxins, gibberellins, cytokinins, and abscisic acid are crucial in regulating multiple aspects of plant growth and development. These metabolites do more than just influence root and shoot growth; they actively participate in shaping root architecture, modulating leaf and flower development, and enhancing the plant’s ability to utilize resources efficiently [299].

Microbial modulation of root architecture, for instance, involves not only stimulating root elongation but also enhancing root branching, which leads to increased root surface area. This improved root system allows plants to access water and nutrients more effectively, particularly in resource-limited environments. Rhizosphere bacteria such as Pseudomonas and Azospirillum secrete IAA (indole-3-acetic acid), which promotes lateral root development and enhances the uptake of mineral nutrients like phosphorus and nitrogen [300,301].

Beyond direct growth promotion, microbial metabolites also modulate other developmental processes, such as flowering time and leaf senescence. Specific rhizobacteria can influence the timing of flowering through ethylene regulation, as seen in Arabidopsis, where microbes alter ethylene synthesis to adjust flowering time based on environmental conditions. This regulation is crucial for synchronizing flowering with favorable conditions, leading to better reproductive success and higher yields. Symbiotic microorganisms, such as AMF, further support nutrient acquisition by facilitating phosphorus uptake, which directly impacts root and shoot biomass [292]. By improving nutrient absorption, these microbes not only enhance overall plant vigor but also allow crops to maintain a balanced nutrient profile, promoting sustainable productivity even under nutrient-poor soil conditions [302]. Root microbial communities also contribute to enhanced mineral homeostasis, particularly for essential elements like iron. Rhizobacteria alter the secretion of root exudates, such as coumarins, that help mobilize iron in the rhizosphere, making it available for plant uptake. These interactions help crops maintain mineral balance, which is critical for healthy growth and stress resilience [303].

### 4.2. Impact on Phenotypic Traits

Microbial metabolites have a substantial influence on crop phenotypic traits, including morphological features, color, flavor, aroma, and nutritional composition. By modifying metabolic pathways, these metabolites can enhance desirable traits, making crops more appealing for both consumers and producers [304].

One of the key ways that rhizosphere microorganisms affect phenotypic traits is through the production of secondary metabolites such as flavonoids, carotenoids, and terpenoids, which contribute to color and aroma in fruits and vegetables [305]. For example, beneficial bacteria that induce the phenylpropanoid pathway in plants lead to enhanced synthesis of anthocyanins, which are responsible for vibrant red, blue, and purple colors in fruits like grapes and berries [306]. This not only improves the visual appeal of crops but also enhances their nutritional quality due to the antioxidant properties of anthocyanins. Flavonoids, produced as a response to microbial elicitation, contribute to improved flavor profiles in crops like tomatoes and peppers. The increased production of these compounds is associated with a more robust antioxidant defense system, which also extends shelf life and enhances post-harvest quality [307]. Similarly, microbial stimulation of terpenoid biosynthesis results in improved aromatic properties in herbs like basil and mint, adding significant value to these crops in culinary markets [308].

In addition to sensory attributes, microbial metabolites also enhance the nutritional content of crops. Rhizosphere microbes like Azotobacter and Bacillus were shown to increase the production of vitamins such as vitamin C and B-complex vitamins in leafy vegetables. These nutritional enhancements make crops more valuable in addressing dietary deficiencies and promoting health among consumers [309].

## 5. Enhancement of Functional Components by Microbial Metabolites

### 5.1. Biosynthesis of Nutraceuticals and Bioactive Compounds

Rhizosphere microbial metabolites have the unique ability to influence plant metabolism and stimulate the biosynthesis of various functional components that are critical for human health, including vitamins, polyphenols, flavonoids, and other antioxidants. These bioactive compounds have gained significant attention for their roles in enhancing plant resilience and their nutritional value as functional foods [310]. For instance, microbial inoculation with beneficial bacteria such as Azospirillum and Bacillus can enhance the accumulation of vitamins like vitamins B and C, which are essential for plant growth and human nutrition. Similarly, the presence of rhizobacteria that produce growth-promoting hormones can lead to an increase in the synthesis of carotenoids and flavonoids—compounds known for their antioxidant and anti-inflammatory properties [311].

The production of these functional components is often regulated through specific molecular pathways influenced by microbial interactions [312]. For example, polyphenol biosynthesis in plants is enhanced by the phenylpropanoid pathway, which can be upregulated by microbial signaling molecules such as salicylic acid and jasmonic acid. Beneficial microbes can trigger these pathways, leading to increased production of flavonoids and other phenolic compounds that serve as defense mechanisms for plants and health-promoting compounds for humans [305].

Biofortification, which aims to enhance the nutritional value of crops, is another promising area where rhizosphere microbial metabolites have a substantial impact. By modulating plant metabolic processes, rhizobacteria can increase the concentration of micronutrients like iron and zinc in edible plant parts, addressing micronutrient deficiencies in human populations [313]. Microbes such as Pseudomonas and Rhizobium have been used in biofortification strategies to enhance iron uptake through the production of siderophores—compounds that bind and transport iron—thereby improving the nutritional content of crops [314].

### 5.2. Microbial Influence on Metabolic Pathways

Rhizosphere microorganisms influence plant metabolic pathways through complex interactions that lead to the increased production of essential oils, amino acids, and other valuable secondary metabolites. These interactions often involve signaling processes that modify the plant’s gene expression and enzymatic activity, which in turn alters the synthesis of specific compounds.

Microbial influence on metabolic pathways can lead to enhanced production of essential oils, which are known for their antimicrobial and therapeutic properties. For instance, inoculation with certain Trichoderma and Pseudomonas species was shown to boost the production of terpenoids and essential oils in aromatic plants like mint and basil [315,316]. These secondary metabolites not only contribute to the plant’s defense but also have significant value in the pharmaceutical, cosmetic, and food industries [317].

Additionally, rhizobacteria such as Bacillus subtilis and Streptomyces can enhance amino acid production by increasing the availability of nitrogen and influencing the plant’s nitrogen assimilation pathways [318]. Amino acids such as lysine, methionine, and tryptophan are not only essential for plant growth but are also important for human nutrition. Through their influence on nitrogen-fixing processes, these microbes help increase amino acid content in crops, making them more nutritionally valuable.

Case studies have demonstrated the successful use of microbial inoculants to improve crop quality through metabolic pathway modulation. For example, inoculation with Rhizobium in legumes was shown to significantly enhance isoflavonoid production, which plays a role in plant defense and offers health benefits as phytoestrogens in human diets [319]. Similarly, the use of Azospirillum has led to increased production of aromatic compounds in fruits such as strawberries, enhancing flavor and market value [320].

Furthermore, microbial elicitors, such as lipo-chitooligosaccharides produced by certain rhizobacteria, were found to stimulate the accumulation of valuable metabolites like alkaloids and saponins. These compounds have pharmaceutical properties, such as anti-inflammatory and anticancer effects, which are beneficial for human health [321]. For instance, studies have shown that the inoculation of medicinal plants with Bacillus species can enhance the production of alkaloids like berberine, which has potent medicinal properties [322]. In addition to direct interactions, microbes also promote changes in the rhizosphere environment that indirectly influence metabolic pathways. The secretion of organic acids by microbes can alter soil pH and increase the bioavailability of nutrients, which in turn impacts plant metabolic activities. By improving nutrient uptake, these changes enhance the synthesis of metabolites that are important for both plant defense and human nutrition.

## 6. Applications in Sustainable Agriculture

### 6.1. Microbial Inoculants and Biofertilizers

The use of microbial inoculants, including biofertilizers and biostimulants, represents a promising approach for enhancing crop performance and soil health in sustainable agriculture. Microbial inoculants introduce beneficial microorganisms into the soil, which helps improve nutrient availability, boost plant growth, and provide resilience against environmental stress [323]. Advances in microbial technologies are making these tools more accessible for farmers seeking sustainable alternatives to chemical fertilizers and pesticides.

Biofertilizers are one of the most widely used forms of microbial inoculants [324]. These products utilize specific microorganisms to convert unavailable nutrients into forms that plants can readily absorb, thereby enhancing soil fertility and promoting plant growth. For instance, nitrogen-fixing bacteria such as Rhizobium, Azotobacter, and Azospirillum are capable of converting atmospheric nitrogen into ammonia, making it available for plant uptake [325]. In leguminous crops, Rhizobium forms a symbiotic relationship with plant roots, producing root nodules where nitrogen is fixed and converted into proteins and other nitrogen-containing compounds essential for plant growth [326].

Biofertilizers also include phosphate-solubilizing microorganisms such as Bacillus and Aspergillus, which produce organic acids to solubilize insoluble phosphates in the soil [327]. This increases phosphorus availability, a critical nutrient for root development and overall plant health. Potassium-solubilizing bacteria and photosynthetic bacteria are also part of the biofertilizer portfolio, offering solutions to mobilize potassium and enhance photosynthetic efficiency, respectively.

Mycorrhizal fungi are another vital component of biofertilizers. These fungi form mutualistic relationships with plant roots, extending the root network and improving water and nutrient absorption, particularly phosphorus [328]. Mycorrhizal inoculants have been effectively used to enhance crop resilience in nutrient-poor soils, making them a valuable tool in sustainable farming practices [329].

Biopesticides represent another application of microbial metabolites in agriculture, helping to reduce the reliance on chemical pesticides. Certain microorganisms produce toxins, antibiotics, or antifungal compounds that target specific pests and pathogens while being harmless to beneficial organisms and humans [330]. For example, Bacillus thuringiensis (Bt) produces insecticidal proteins that target a range of insect pests, making it a popular choice for integrated pest management [331]. Actinomycetes, such as Streptomyces, produce antifungal compounds that are effective against plant pathogens like those causing tomato blight and cucumber wilt, offering a natural means of disease control [332].

Microbial metabolites are also employed as biostimulants, which promote plant growth by enhancing nutrient uptake, root growth, and stress tolerance. Compounds like auxins, gibberellins, and cytokinins produced by rhizobacteria stimulate plant hormone pathways, leading to enhanced root and shoot development [333]. These biostimulants also improve crop resilience to abiotic stresses, such as drought and salinity, by modulating physiological responses, including osmotic adjustment and antioxidant activity.

In practice, microbial fertilizers can be categorized based on the type of microorganisms they contain—bacterial fertilizers, fungal fertilizers, and actinomycete fertilizers. Bacterial fertilizers, for example, include nitrogen-fixing and phosphate-solubilizing strains that improve nutrient availability [334,335]. Actinomycetes are particularly important, as they are responsible for producing approximately 70% of microbial secondary metabolites, many of which play a role in plant growth promotion and disease control. Fungal fertilizers, such as mycorrhizal inoculants, help improve plant hormone production and nutrient uptake, further supporting sustainable crop production [336,337].

### 6.2. Challenges and Future Perspectives

Despite the numerous benefits offered by microbial inoculants and biofertilizers, several challenges must be addressed to ensure their widespread adoption and effectiveness in sustainable agriculture. One major challenge is the variability in field conditions, which can significantly affect the performance of microbial inoculants. Factors such as soil type, pH, temperature, moisture, and nutrient availability influence the activity and survival of introduced microorganisms. Therefore, field-specific trials are essential to determine the efficacy of microbial products under different conditions, ensuring optimal results.

Another challenge is the sensitivity of microbial metabolites to environmental conditions. Microbial metabolites are biologically active substances that require suitable environmental conditions—including appropriate levels of water, nutrients, temperature, and aeration—to function effectively. Environmental stressors such as high temperatures, low moisture, or the presence of competing microorganisms can limit the effectiveness of microbial inoculants. Therefore, careful management of soil conditions and adherence to best practices for microbial application are critical for success.

Additionally, some microbial secondary metabolites can have adverse or even toxic effects on plants when applied improperly or in excess. For instance, metabolites that alter hormone levels must be carefully regulated to avoid unintended effects such as stunted growth or premature flowering. The need for proper application techniques and adherence to recommended dosages cannot be overstated, as misuse may lead to reduced efficacy or unintended negative impacts on crop health.

In the production and use of microbial metabolites, the following considerations are crucial:

Tailored Application: The selection of suitable microbial inoculants must be based on the specific requirements of the crop, soil characteristics, and environmental conditions. Applying the right microbial product for a particular crop and condition is key to maximizing benefits and avoiding potential negative effects.

Optimal Application Practices: It is essential to apply microbial products under conditions that support their activity. For example, soil moisture should be adequate to support microbial survival, and care should be taken to avoid co-application with incompatible substances, such as fungicides or unprocessed organic manure, which may inhibit microbial activity.

Product Information and Awareness: Farmers and agricultural practitioners must be informed about the specific characteristics and requirements of microbial products. Clear product information, including application guidelines and environmental requirements, will help ensure that these products are used effectively to achieve the desired results.

Future Perspectives: To overcome the challenges of variability and environmental sensitivity, future research should focus on the development of robust microbial strains that can withstand a range of environmental stresses, including extreme temperatures, pH variations, and soil salinity. The development of microbial consortia—combinations of complementary microbial species that work synergistically—presents an exciting opportunity to improve the resilience and efficacy of microbial inoculants. Such consortia could provide more consistent performance across diverse field conditions, reducing the variability in outcomes.

The integration of advanced technologies, such as metagenomics and synthetic biology, offers promising avenues for optimizing microbial inoculants. Metagenomics can help identify beneficial microbial communities present in specific soil environments, while synthetic biology can be used to engineer microbial strains with enhanced traits, such as increased metabolite production or improved environmental tolerance.

Ultimately, the successful application of microbial metabolites and inoculants in sustainable agriculture requires a holistic approach that considers soil health, crop requirements, environmental conditions, and the characteristics of the microbial products. By addressing these challenges and advancing our understanding of microbial–plant interactions, microbial inoculants have the potential to transform agriculture, making it more productive, sustainable, and resilient to future challenges.

## 7. Conclusions

Rhizosphere microbial metabolites play a crucial role in promoting plant health, growth, and resilience, making them integral components of sustainable agricultural practices. These metabolites, including phytohormones, antibiotics, volatile organic compounds (VOCs), and exopolysaccharides (EPS), significantly enhance nutrient uptake, stimulate root and shoot development, and protect crops from both biotic and abiotic stresses. Their application as microbial inoculants, biofertilizers, biostimulants, and biopesticides provides multiple benefits, including improved soil fertility, enhanced plant growth, and natural pest and disease management, ultimately reducing the reliance on synthetic fertilizers and pesticides.

However, the potential of microbial metabolites extends beyond individual benefits. They contribute to a holistic approach to crop management, supporting nutrient cycling, enhancing stress resilience, and improving crop productivity and quality. Despite the challenges related to environmental variability and the sensitivity of microbial products to field conditions, ongoing advancements in developing robust microbial strains, microbial consortia, and innovative application techniques hold promise for overcoming these limitations.

To achieve effective and sustainable outcomes, a deeper understanding of plant–microbe interactions and field-specific adaptations is essential. By harnessing the power of microbial metabolites, agricultural systems can become more resilient, productive, and environmentally friendly, contributing to global food security and the long-term sustainability of agricultural practices.

## Figures and Tables

**Table 2:** Hormones and functions of common microbial sources.

Types of Growth Regulators	Structure	Microbes Involved	Research Example and Impact on Plants	References
Auxins (Indole-3-Acetic Acid, IAA)	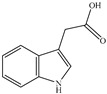	*Chryseobacterium* culicis, *Paenibacillus* polymyxa; *Bacillus* safensis 33C, *Rhodococcus* rhodochrous YZ, *Corynebacterium* stationis 29B; *Azospirillum* spp.; *Methylobacterium* symbioticum, *Bacillus* spp., *Streptomyces* sp., *Pseudomonas* mandelii IB-Ki14	Auxin-producing bacteria promote barley rhizosheath formation; IAA accumulation benefited the germination and early vegetative growth of tomatoes; *Azospirillum* spp. produce IAA, promoting root elongation and increasing nutrient uptake in crops like rice and wheat.	[79,80,81,82]
Gibberellins (GA)	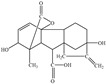	*Rhizobia*, *Pseudomonas* spp., *Azospirillum* spp., *Bacillus* spp, *Bradyrhizobium* diazoefficiens., *Fusarium* fujikuroi; *Gibberella* fujikuroi	Production of the plant hormone gibberellin by rhizobia increases host legume nodule size.	[83,84]
Cytokinins (CTK)	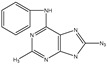	*Bacillus* subtilis IB-22, *Pseudomonas* fluorescens G20-18, *Bacillus* toyonensis, *Rhodobacter* sphaeroides, *Actinomycetes*	Ability to promote growth and increase tolerance to drought stress in tomato; promotes corn growth and enhances root development.	[85,86,87,88,89]
abscisic acid (ABA)	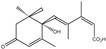	*Bradyrhizobium* japonicum BRC 2485, *Cercospora*, *Alternaria*, *Botrytis*, *Penicillium*, *Ceratocystis* and *Aspergillus*	Regulation of plant growth and development and induction of plant resistance to adverse growing conditions.	[90,91,92,93]
Ethylene (ETH)	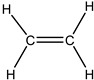	*Rhodospirillum* rubrum	Regulates plant growth and development and response to environmental stresses.	[94]
Salicylate (SA)	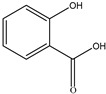	*Streptomyces* sp. JCK-8055; *Pseudomonas* syringae pv. actinidiae (*Psa* biovar 3); *Escherichia* colityr002; *Pseudomonas* aeruginosa; *Trichasporon* moniliiforme	Additionally, JCK-8055 can produce the plant growth regulation hormone indole-3-acetic acid (IAA) and hydrolytic enzymes, including protease, gelatinase, and cellulase. JCK-8055 treatment also triggered the expression of salicylate (SA) and jasmonate (JA) signaling pathway marker genes, such as PR1, PR2, and PR3.	[95,96,97]
Jasmonate (JA)		*Lasiodiplodia* theobromae; *Botryodiplodia* theobromae; *Gibberella* fujikuroi; *Diplodia* gossypina; *Bacillus* velezensis A-27; *Stagonospora* nodorum (Berk.)	Activation of defense mechanisms against plant pathogens and injuries.	[96,98,99,100,101,102,103]
Pironetin	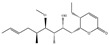	*Streptomyces* sp. NK10958	Plant-growth-regulating activity, immunosuppressive effect and significant antitumor activity, such as, nhibition of crop plant height increase.	[104,105]
Oxamycin(cyoloserin)	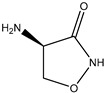	*Kocuria* rhizophila PT10	Regulates plant growth and increases the sugar content of plantains;Effective antagonism against the phytopathogenic fungi *Fusarium* gray mold BC21 and *Fusarium* graminearum g1.	[106]

**Table 3:** Different types of lipopeptides produced by bacteria and their effects on plant pathogens.

Type	Sources	Functions	References
Surface Activator (Surfactin)	*Bacillus* subtitles *Bacillus* velezensis *Bacillus* polymyxa	In the fight against plant pathogens, Surfactin exhibits broad-spectrum antimicrobial activity, with significant inhibition of a wide range of pathogenic fungi, including *Fusarium* moniliforme.	[156,157,158]
Fengycin	*Bacillus* subtitles *Bacillus* polymyxa	good fungistatic activity, especially effective against filamentous fungi.	[159,160,161,162]
Bacillomycin (Bacillocin)	*Bacillus* sp.	exhibit potent inhibitory effects against a wide range of bacteria and fungi.	[163,164,165,166]
Iturin	*Bacillus* subtitles *Bacillus* cereus *B.* velezensis *B. Amyloliquefaciens* *Bacillus* polymyxa	Iturin has a preventive and control effect on cucumber bacterial leaf spot and soft rot, and wheat blast disease.	[167,168,169,170]
polymyxins	*Bacillus* polymyxa	It has broad-spectrum antimicrobial activity, can promote plant growth and improve soil fertility, and can be used in microbial pesticides.	[171,172,173,174]

**Table 4:** Common types and sources of antibiotics of microbial origin and their functions.

Type	Species	Structure	Sources	Functions	References
peptide antibiotic	Bacillomycin D	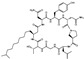	*Bacillus* subtitles *Bacillus* cereus *B.* velezensis *B.* Amyloliquefaciens	Strong inhibitory activity against *Aspergillus* flavus, *Fusarium* graminearum, *Anthrax*, *Staphylococcus* aureus*, Rhizoctonia* solani, etc.	[176] [177] [32,178,179,180,181,182,183,184,185]
	Enduracidins	C_108_H_140_Cl_2_N_26_O_31_	*S.* fungicidicus	Good inhibitory activity against most Gram-positive bacteria.	[186,187,188,189,190]
phenoxazine antibiotic	Phenazine-1-carboxylic acid, PCA	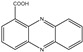	*Pseudomonas aeruginosa* M18	PCA is effective in the control of rice blight, watermelon blight, bell pepper blight, chili pepper blight and cucumber blight.	[191,192,193]
	Phenazine-1-carboxamide, PCN	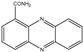	*P. Chlororaphis* and *P. aeruginosa*	PCN has significant antagonistic effects against a variety of phytopathogenic fungi such as *Fusarium* spinosum, *Xanthomonas campestris*, *Fusarium* verticillioides, *Fusarium* tomatitum, and *Fusarium* ultimateis.	[194,195,196,197]
	2-hydroxyphenazines, 2-OH-PHZ	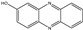	*P.chlororaphis* 30–84 Q.and *P. chlororaphis* GP72	Broad-spectrum fungicide, can effectively prevent and control wheat total eclipse disease, for the epidemic mold, mildew and other plant pathogenic fungi also has a very good inhibition effect.	[198,199,200]
Polyketide antibiotics	Borrelidin	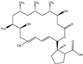	*Streptomyces* spp.	Plant pathogenic fungi such as *Mycosphaerella* sojae, *Mycosphaerella* melongena, *Mycosphaerella* terminalis, and *Mycosphaerella* peppers have significant antagonistic effects.	[201]
	Milbemycins	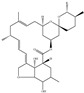	*Streptomyces* bingchenggensis	Possesses potent anthelmintic and insecticidal activity.	[202,203,204,205]
	Pyoluteorin, Plt	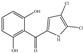	*P.* aeruginosa	Broad-spectrum antimicrobial activity against fungi (especially *Aspergillus* spp.) and bacteria.	[192,206,207,208]
	Anisomycin	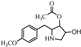	*S.* roseochromogenes *S.* griseolus	Control of fungal diseases of crops, such as crop powdery mildew, watermelon rot, rice blight, etc.	[209]
	Xenocoumacin 1	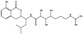	*Xenorhabdus* nematophila	Strong inhibitory activity against *Streptomyces* intermedius, *Aspergillus*, *Rickettsia*, *Mycobacterium* difficile, etc.	[210,211,212]
nucleoside antibiotic	Xinaomycin	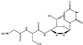	*Streptomyces* norvegicus Xi Ao-3	Strong inhibitory activity against *Botrytis* cinerea, anthracnose, cotton wilt, tomato mosaic virus and watermelon mosaic virus.	[213]
avermectin		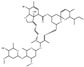	*Streptomyces* avermitilis	Insecticidal, acaricidal and nematicidal activity.	[214,215]

**Table 5:** Kinds of VOC-producing microorganisms and the functions of VOCs.

Microbial Species	Strains	Functions	References
Bacteria	*Streptomyces* globisporus JK-1	Inhibition of conidial germination and mycelial growth.The abnormality of conidia and mycelium morphology.	[222,223,224,225]
	*Streptomyces* yanglinensis 3-10	Inhibition of the disease and the generation of aflatoxin.Exhibited high antifungal activity against *Staphylococcus* griseus, *Trichoderma*, *Rhizopus* stolonifera and *Botrytis* cinerea.	[226,227,228]
	*Streptomyces* philanthi RM-1-138	Antagonistic activity against phytopathogenic fungi, e.g., anthracnose of chili peppers, rice blight, etc.	[229,230,231,232]
	*Bacillus* subtilis Y13	It has good control effect on many kinds of pathogenic bacteria, such as anthracnose of oil tea.	[233]
	*Pseudomonas* fluorescens UM16, UM240, UM256, UM270	Exhibits high antagonism against the plant pathogen *Staphylococcus* griseus.	[234,235,236,237]
	*Enterobacter* asburiae Vt-7	Blocks the production of aflatoxins and protects against fungal pathogens and mycotoxins in foods and grains.	[238]
	*Staphylococcus sciuri* MarR44	Antifungal activity against strawberry anthracnose.	[239]
Polycellularis fungus	*Muscodor* brasiliensis sp. LGMF1255, LGMF1256	Inhibition of the plant pathogen *P.* digitatum.	[240,241]
	*Nodulisporium* spp. CMU-UPE34	Inhibits or kills a variety of plant pathogens, such as post-harvest decay of citrus fruit.	[242,243,244]
	*Wickerhamomyces* anomalus, *Metschnikowia* pulcherrima, *Saccharomyces* cerevisiae	Inhibits the growth of rot-causing fungi and controls post-harvest diseases of strawberries.	[245,246,247,248,249]
	*Candida* intermedia C410	Controlling post-harvest gray mold in strawberries; As a food antimicrobial agent.	[250,251]

## Data Availability

The data that support the findings of this study are available from the corresponding author upon reasonable request.

## References

[1] Yuan Y., Zuo J., Zhang H., Zu M., Liu S. (2022). The Chinese medicinal plants rhizosphere: Metabolites, microorganisms, and interaction. Rhizosphere.

[2] Cao T., Fang Y., Chen Y., Kong X., Yang J., Alharbi H., Kuzyakov Y., Tian X. (2022). Synergy of saprotrophs with mycorrhiza for litter decomposition and hotspot formation depends on nutrient availability in the rhizosphere. Geoderma.

[3] Vandana U.K., Rajkumari J., Singha L.P., Satish L., Alavilli H., Sudheer P.D.V.N., Chauhan S., Ratnala R., Satturu V., Mazumder P.B. (2021). The Endophytic Microbiome as a Hotspot of Synergistic Interactions, with Prospects of Plant Growth Promotion. Biology.

[4] Peng Z., Guo X.-Z., Xu Y., Liu D.-H., Wang H.-Y., Guo L.-P., Zhang Y. (2020). Advances in interaction between medicinal plants and rhizosphere microorganisms. Zhongguo Zhong Yao Za Zhi = Zhongguo Zhongyao Zazhi = China J. Chin. Mater. Medica.

[5] Che J., Wu Y., Yang H., Chang Y., Wu W., Lyu L., Wang X., Cao F., Li W. (2024). Metabolites of blueberry roots at different developmental stages strongly shape microbial community structure and intra-kingdom interactions at the root-soil interface. Sci. Total Environ..

[6] Dahlstrom K.M., McRose D.L., Newman D.K. (2020). Keystone metabolites of crop rhizosphere microbiomes. Curr. Biol..

[7] Shahriar S.A., Islam M.N., Chun C.N.W., Kaur P., Rahim M.A., Islam M.M., Uddain J., Siddiquee S. (2022). Microbial Metabolomics Interaction and Ecological Challenges of *Trichoderma* Species as Biocontrol Inoculant in Crop Rhizosphere. Agronomy.

[8] Zhuang Y., Wang H., Tan F., Wu B., Liu L., Qin H., Yang Z., He M. (2024). Rhizosphere metabolic cross-talk from plant-soil-microbe tapping into agricultural sustainability: Current advance and perspectives. Plant Physiol. Biochem..

[9] Korenblum E., Dong Y., Szymanski J., Panda S., Jozwiak A., Massalha H., Meir S., Rogachev I., Aharoni A. (2020). Rhizosphere microbiome mediates systemic root metabolite exudation by root-to-root signaling. Proc. Natl. Acad. Sci. USA.

[10] Moutia J.-F.Y., Saumtally S., Spaepen S., Vanderleyden J. (2010). Plant growth promotion by *Azospirillum* sp. in sugarcane is influenced by genotype and drought stress. Plant Soil.

[11] Ledger T., Rojas S., Timmermann T., Pinedo I., Poupin M.J., Garrido T., Richter P., Tamayo J., Donoso R. (2016). Volatile-Mediated Effects Predominate in *Paraburkholderia phytofirmans* Growth Promotion and Salt Stress Tolerance of *Arabidopsis thaliana*. Front. Microbiol..

[12] Dandurishvili N., Toklikishvili N., Ovadis M., Eliashvili P., Giorgobiani N., Keshelava R., Tediashvili M., Vainstein A., Khmel I., Szegedi E. (2011). Broad-range antagonistic rhizobacteria *Pseudomonas fluorescens* and *Serratia plymuthica* suppress *Agrobacterium* crown gall tumours on tomato plants. J. Appl. Microbiol..

[13] Ghanem G.A.M., Gebily D.A.S., Ragab M.M., Ali A.M., Soliman N.E.-D.K., Abd El-Moity T.H. (2022). Efficacy of antifungal substances of three *Streptomyces* spp. against different plant pathogenic fungi. Egypt. J. Biol. Pest Control.

[14] Haidar R., Fermaud M., Calvo-Garrido C., Roudet J., Deschamps A. (2016). Modes of action for biological control of *Botrytis cinerea* by antagonistic bacteria. Phytopathol. Mediterr..

[15] Patni B., Bhattacharyya M., Pokhriyal A. (2023). The role of signaling compounds in enhancing rice allelochemicals for sustainable agriculture: An overview. Planta.

[16] Duan G., Li C., Liu Y., Ma X., Luo Q., Yang J. (2021). *Magnaporthe oryzae* systemic defense trigger 1 (MoSDT1)-mediated metabolites regulate defense response in Rice. BMC Plant Biol..

[17] Wang L., Huang X., Li J., Huang J., Bao S., He C., Zhang M., Xiang T. (2022). Metabolites of zearalenone and phytohormones secreted by endophytic fungus strain TH15 regulating the root development in *Tetrastigma hemsleyanum*. Plant Cell Tissue Organ Cult..

[18] Boak E.N., Kirolos S., Pan H., Pierson L.S., Pierson E.A. (2022). The Type VI Secretion Systems in Plant-Beneficial Bacteria Modulate Prokaryotic and Eukaryotic Interactions in the Rhizosphere. Front. Microbiol..

[19] Liu C., Yu J., Ying J., Zhang K., Hu Z., Liu Z., Chen S. (2023). Integrated metagenomics and metabolomics analysis reveals changes in the microbiome and metabolites in the rhizosphere soil of *Fritillaria unibracteata*. Front. Plant Sci..

[20] Nihorimbere V., Ongena M., Smargiassi M., Thonart P. (2011). Beneficial effect of the rhizosphere microbial community for plant growth and health. Biotechnol. Agron. Soc. Environ..

[21] Kumar U., Raj S., Sreenikethanam A., Maddheshiya R., Kumari S., Han S., Kapoor K.K., Bhaskar R., Bajhaiya A.K., Gahlot D.K. (2023). Multi-Omics Approaches in Plant-Microbe Interactions Hold Enormous Promise for Sustainable Agriculture. Agronomy.

[22] Cui Q.L., Beiyuan J., Chen Y.L., Li M.D., Qiu T.Y., Zhao S.L., Zhu X.Z., Chen H.S., Fang L.C. (2024). Synergistic enhancement of plant growth and cadmium stress defense by *Azospirillum brasilense* and plant heme: Modulating the growth—Defense relationship. Sci. Total Environ..

[23] Perez-Fernandez M., De Lara-Del Rey I.A., Magadlela A. (2024). Shining a Light on Symbiosis: N-Fixing Bacteria Boost Legume Growth under Varied Light Conditions. Agriculture.

[24] Rieusset L., Rey M., Muller D., Vacheron J., Gerin F., Dubost A., Comte G., Prigent-Combaret C. (2020). Secondary metabolites from plant-associated *Pseudomonas* are overproduced in biofilm. Microb. Biotechnol..

[25] Xu Y., Zhu M., Feng Y., Xu H. (2023). *Panax notoginseng*-microbiota interactions: From plant cultivation to medicinal application. Phytomedicine.

[26] Lee Y.H., Jang S.J., Han J.-H., Bae J.S., Shin H., Park H.J., Sang M.K., Han S.H., Kim K.S., Han S.-W. (2018). Enhanced Tolerance of Chinese Cabbage Seedlings Mediated by *Bacillus aryabhattai* H26-2 and *B-siamensis* H30-3 against High Temperature Stress and Fungal Infections. Plant Pathol. J..

[27] Bomfeti C.A., Florentino L.A., Guimaraes A.P., Cardoso P.G., Guerreiro M.C., de Souza Moreira F.M. (2011). Exopolysaccharides produced by the symbiotic nitrogen-fixing bacteria of leguminosae. Rev. Bras. Cienc. Solo.

[28] Sher Y., Baker N.R., Herman D., Fossum C., Hale L., Zhang X., Nuccio E., Saha M., Zhou J., Pett-Ridge J. (2020). Microbial extracellular polysaccharide production and aggregate stability controlled by switchgrass (*Panicum virgatum*) root biomass and soil water potential. Soil Biol. Biochem..

[29] Kaushal P., Ali N., Saini S., Pati P.K., Pati A.M. (2023). Physiological and molecular insight of microbial biostimulants for sustainable agriculture. Front. Plant Sci..

[30] Staropoli A., Di Mola I., Ottaiano L., Cozzolino E., Pironti A., Lombardi N., Nanni B., Mori M., Vinale F., Woo S.L. (2024). Biodegradable Mulch Films and Bioformulations Based on *Trichoderma* sp. and Seaweed Extract Differentially Affect the Metabolome of Industrial Tomato Plants. J. Fungi.

[31] Bhat B.A., Tariq L., Nissar S., Islam S.T., Ul Islam S., Mangral Z., Ilyas N., Sayyed R.Z., Muthusamy G., Kim W. (2022). The role of plant-associated rhizobacteria in plant growth, biocontrol and abiotic stress management. J. Appl. Microbiol..

[32] An B., Du D., Huang Z., Pu Z., Lv J., Zhu L., Liu S., Zhang L., Chen G., Lu L. (2024). Biocontrol of citrus fungal pathogens by lipopeptides produced by *Bacillus velezensis* TZ01. Front. Microbiol..

[33] Das J., Panigrahy M., Mohanty S., Jena B., Nayak R.K., Shukla A.K. (2024). Plant Growth-Promoting Microbes for Sustainable Crop Production (a Review). Appl. Biochem. Microbiol..

[34] Su L., Zhang J., Fan J., Li D., Zhao M., Wang Y., Pan H., Zhao L., Zhang X. (2024). Antagonistic Mechanism Analysis of *Bacillus velezensis* JLU-1, a Biocontrol Agent of Rice Pathogen *Magnaporthe oryzae*. J. Agric. Food Chem..

[35] Wang T., Li W., Wang F., Li J., Qin J., Song Z., Xu J., Qiu H., Cheng Y. (2024). Biocontrol potential of Bacillus velezensis SEC-024A against southern blight of industrial hemp. Ind. Crops Prod..

[36] Yang K., Dai X., Maitikadir Z., Zhang H., Hao H., Yan C. (2024). Comparative genome analysis of endophytic *Bacillus amyloliquefaciens* MR4: A potential biocontrol agent isolated from wild medicinal plant root tissue. J. Appl. Genet..

[37] Wang X., Wang A., Zhuang M., Ke S., Ning M., Zhou Z. (2024). Impact of metabolites derived from *Bacillus velezensis* on the germination of tigernut seeds and the underlying molecular regulatory mechanism. Food Biosci..

[38] Bernabe-Perez E.A., Gaytan P., Juarez-Gonzalez V.R., Hernandez-Garcia I.J., Tapia-Pastrana G., Quintero-Hernandez V., Martinez-Martinez L.L. (2024). Heterologous Production of Bacteriocin EMM1 from *Pseudomonas Protegens* and its Antimicrobial Activity against Multidrug-resistant Clinical Isolates. Int. J. Pept. Res. Ther..

[39] Li C., Gao X., Huo Y., Asseri T.A.Y., Tian X., Luo K. (2024). Evaluation of biocontrol efficacy of rhizosphere *Pseudomonas aeruginosa* for management of *Phytophthora capsici* of pepper. PLoS ONE.

[40] Stepanov A.A., Shulaev N.A., Vasilchenko A.S. (2024). The Ecological Strategy Determines the Response of Fungi to Stress: A Study of the 2,4-diacetylphloroglucinol Activity Against *Aspergillus* and *Fusarium* Species. J. Basic Microbiol..

[41] Tuerdibieke M., Tian X., An X., Feng Y., Liu W. (2024). Isolation and identification of endophytic fungi from Alhagi sparsifolia Shap. and their antibacterial activity. Heliyon.

[42] Chen X., Liu J., Chen A.J., Wang L., Jiang X., Gong A., Liu W., Wu H. (2024). Burkholderia ambifaria H8 as an effective biocontrol strain against maize stalk rot via producing volatile dimethyl disulfide. Pest Manag. Sci..

[43] Li W., Fu Y., Jiang Y., Hu J., Wei Y., Li H., Li J., Yang H., Wu Y. (2024). Synergistic Biocontrol and Growth Promotion in Strawberries by Co-Cultured *Trichoderma harzianum* TW21990 and *Burkholderia vietnamiensis* B418. J. Fungi.

[44] Wang D.-d., Nie J.-j., Zhao R.-b., Lu J., Wei Y.-x., Yu L., Chen F.-f., Pan Y.-m. (2024). A novel *Burkholderia pyrrocinia* strain effectively inhibits *Fusarium graminearum* growth and deoxynivalenol (DON) production. Pest Manag. Sci..

[45] Keswani C., Mishra S., Sarma B.K., Singh S.P., Singh H.B. (2014). Unraveling the efficient applications of secondary metabolites of various *Trichoderma* spp.. Appl. Microbiol. Biotechnol..

[46] Khan R.A.A., Najeeb S., Mao Z., Ling J., Yang Y., Li Y., Xie B. (2020). Bioactive Secondary Metabolites from *Trichoderma* spp. against Phytopathogenic Bacteria and Root-Knot Nematode. Microorganisms.

[47] Li M.-F., Li G.-H., Zhang K.-Q. (2019). D Non-Volatile Metabolites from *Trichoderma* spp.. Metabolites.

[48] Onufrak A.J., Gazis R., Gwinn K., Klingeman W., Khodaei S., Onate L.I.P., Finnell A., Givens S., Chen C., Holdridge D.R. (2024). Potential biological control agents of *Geosmithia morbida* restrict fungal pathogen growth via mycoparasitism and antibiosis. BioControl.

[49] Zeilinger S., Gruber S., Bansal R., Mukherjee P.K. (2016). Secondary metabolism in *Trichoderma*—Chemistry meets genomics. Fungal Biol. Rev..

[50] Zin N.A., Badaluddin N.A. (2020). Biological functions of *Trichoderma* spp. for agriculture applications. Ann. Agric. Sci..

[51] Lukassen M.B., Saei W., Sondergaard T.E., Tamminen A., Kumar A., Kempken F., Wiebe M.G., Sorensen J.L. (2015). Identification of the Scopularide Biosynthetic Gene Cluster in *Scopulariopsis brevicaulis*. Mar. Drugs.

[52] Mou X.-F., Liu X., Xu R.-F., Wei M.-Y., Fang Y.-W., Shao C.-L. (2018). Scopuquinolone B, a new monoterpenoid dihydroquinolin-2(1*H*)-one isolated from the coral-derived *Scopulariopsis* sp fungus. Nat. Prod. Res..

[53] Moubasher H., Elkholy A., Sherif M., Zahran M., Elnagdy S. (2022). In Vitro Investigation of the Impact of Bacterial-Fungal Interaction on Carbapenem-Resistant *Klebsiella pneumoniae*. Molecules.

[54] Quach N.T., Vu T.H.N., Nguyen T.T.A., Le P.C., Do H.G., Nguyen T.D., Thao P.T.H., Nguyen T.T.L., Chu H.H., Phi Q.-T. (2023). Metabolic and genomic analysis deciphering biocontrol potential of endophytic Streptomyces albus RC2 against crop pathogenic fungi. Braz. J. Microbiol..

[55] Qureshi S.A., Ruqqia, Sultana V., Ara J., Ehteshamul-Haque S. (2012). Nematicidal potential of culture filtrates of soil fungi associated with rhizosphere and rhizoplane of cultivated and wild plants. Pak. J. Bot..

[56] Tang J., Huang X., Cao M.-H., Wang Z., Yu Z., Yan Y., Huang J.-P., Wang L., Huang S.-X. (2022). Mono-/Bis-Alkenoic Acid Derivatives From an Endophytic Fungus *Scopulariopsis candelabrum* and Their Antifungal Activity. Front. Chem..

[57] Yang F., Jiang H., Chang G., Liang S., Ma K., Cai Y., Tian B., Shi X. (2023). Effects of Rhizosphere Microbial Communities on Cucumber *Fusarium* wilt Disease Suppression. Microorganisms.

[58] Ahluwalia V., Kumar J., Rana V.S., Sati O.P., Walia S. (2015). Comparative evaluation of two *Trichoderma harzianum* strains for major secondary metabolite production and antifungal activity. Nat. Prod. Res..

[59] Alwhibi M.S., Hashem A., Abd Allah E.F., Alqarawi A.A., Soliman D.W.K., Wirth S., Egamberdieva D. (2017). Increased resistance of drought by *Trichoderma harzianum* fungal treatment correlates with increased secondary metabolites and proline content. J. Integr. Agric..

[60] Guo R., Li G., Zhang Z., Peng X. (2022). Structures and Biological Activities of Secondary Metabolites from *Trichoderma harzianum*. Mar. Drugs.

[61] Rahman M.A., Begum M.F., Alam M.F. (2009). Screening of Trichoderma Isolates as a Biological Control Agent against Ceratocystis paradoxa Causing Pineapple Disease of Sugarcane. Mycobiology.

[62] Singh A., Gupta R., Srivastava M., Gupta M.M., Pandey R. (2016). Microbial secondary metabolites ameliorate growth, *in planta* contents and lignification in *Withania somnifera* (L.) Dunal. Physiol. Mol. Biol. Plants.

[63] Vinale F., Manganiello G., Nigro M., Mazzei P., Piccolo A., Pascale A., Ruocco M., Marra R., Lombardi N., Lanzuise S. (2014). A Novel Fungal Metabolite with Beneficial Properties for Agricultural Applications. Molecules.

[64] Alarjani K.M., Elshikh M.S. (2024). Plant growth-promoting and biocontrol traits of endophytic *Bacillus licheniformis* against soft rot causing *Pythium myriotylum* in ginger plant. J. Basic Microbiol..

[65] Dhakshinamoorthy M., Packiam K.K., Kumar P.S., Saravanakumar T. (2021). Endophytic fungus Diaporthe caatingaensis MT192326 from Buchanania *axillaris:* An indicator to produce biocontrol agents in plant protection. Environ. Res..

[66] Sebestyen D., Perez-Gonzalez G., Ghoshal M., Goodell B. (2021). Effect of Abamectin on Fungal Growth and Its Efficacy as a Miticide in the Laboratory. Phytopathology.

[67] Yan L., Khan R.A.A. (2021). Biological control of bacterial wilt in tomato through the metabolites produced by the biocontrol fungus, *Trichoderma harzianum*. Egypt. J. Biol. Pest Control.

[68] Carrillo-Flores E., Arreola-Rivera J., Mariana Pazos-Solis D., Bocanegra-Mondragon M., Fierro-Romero G., Elena Mellado-Rojas M., Beltran-Pena E. (2022). Participation of Auxin Transport in the Early Response of the *Arabidopsis* Root System to Inoculation with *Azospirillum brasilense*. Phyton-Int. J. Exp. Bot..

[69] Chen C.-Y., Selvaraj P., Naqvi N.I. (2023). Functional analysis of auxin derived from a symbiotic mycobiont. Front. Plant Sci..

[70] Hayat R., Ali S., Amara U., Khalid R., Ahmed I. (2010). Soil beneficial bacteria and their role in plant growth promotion: A review. Ann. Microbiol..

[71] Sangwan P., Sangwan R., Malik V., Singh M. (2023). Evaluation of antibiotics and bioagents for the management of black rot disease of cabbage and their effect on extracellular polysaccharide secretion. Bangladesh J. Bot..

[72] Tian Z., Du Y., Lu Y., Zhu J., Long C.-a. (2024). Exploration of the antimicrobial activities of biocontrol agent *Streptomyces* strain h114 against *Penicillium digitatum* in citrus. Postharvest Biol. Technol..

[73] Yang Y., Chen R., Rahman M.U., Wei C., Fan B. (2023). The *sprT* Gene of *Bacillus velezensis* FZB42 Is Involved in Biofilm Formation and Bacilysin Production. Int. J. Mol. Sci..

[74] Camaille M., Fabre N., Clement C., Barka E.A. (2021). Advances in Wheat Physiology in Response to Drought and the Role of Plant Growth Promoting Rhizobacteria to Trigger Drought Tolerance. Microorganisms.

[75] Mastny J., Barta J., Kastovska E., Picek T. (2021). Decomposition of peatland DOC affected by root exudates is driven by specific r and K strategic bacterial taxa. Sci. Rep..

[76] Thepbandit W., Srisuwan A., Siriwong S., Nawong S., Athinuwat D. (2023). *Bacillus vallismortis* TU-Orga21 blocks rice blast through both direct effect and stimulation of plant defense. Front. Plant Sci..

[77] Zheng H., Guber A.K., Kuzyakov Y., Zhang W., Kravchenko A.N. (2022). Plant species and plant neighbor identity affect associations between plant assimilated C inputs and soil pores. Geoderma.

[78] Yang Q.L., Zhang H., You J., Yang J., Zhang Q., Zhao J.J., Aimaier R., Zhang J.B., Han S.C., Zhao H.P. (2023). Transcriptome and metabolome analyses reveal that *Bacillus subtilis* BS-Z15 lipopeptides mycosubtilin homologue mediates plant defense responses. Front. Plant Sci..

[79] Xu F., Liao H., Yang J., Zhang Y., Yu P., Cao Y., Fang J., Chen S., Li L., Sun L. (2023). Auxin-producing bacteria promote barley rhizosheath formation. Nat. Commun..

[80] Cai G., Li J., Zhou M., Zhu G., Li Y., Lv N., Wang R., Li C., Pan X. (2022). Compost-derived indole-3-acetic-acid-producing bacteria and their effects on enhancing the secondary fermentation of a swine manure-corn stalk composting. Chemosphere.

[81] de-Bashan L.E., Antoun H., Bashan Y. (2008). Involvement of indole-3-acetic acid produced by the growth-promoting bacterium *Azospirillum* spp. in promoting growth of *Chlorella vulgaris*. J. Phycol..

[82] Pappalettere L., Bartolini S., Toffanin A. (2024). Auxin-Producing Bacteria Used as Microbial Biostimulants Improve the Growth of Tomato (*Solanum lycopersicum* L.) Seedlings in Hydroponic Systems. Biotech.

[83] Nett R.S., Bender K.S., Peters R.J. (2022). Production of the plant hormone gibberellin by rhizobia increases host legume nodule size. Isme J..

[84] Wang H.N., Ke X., Jia R., Huang L.G., Liu Z.Q., Zheng Y.G. (2022). Multivariate modular metabolic engineering for enhanced gibberellic acid biosynthesis in *Fusarium fujikuroi*. Bioresour. Technol..

[85] Akhtyamova Z., Martynenko E., Arkhipova T., Seldimirova O., Galin I., Belimov A., Vysotskaya L., Kudoyarova G. (2023). Influence of Plant Growth-Promoting Rhizobacteria on the Formation of Apoplastic Barriers and Uptake of Water and Potassium by Wheat Plants. Microorganisms.

[86] Mekureyaw M.F., Pandey C., Hennessy R.C., Nicolaisen M.H., Liu F., Nybroe O., Roitsch T. (2022). The cytokinin-producing plant beneficial bacterium *Pseudomonas fluorescens* G20-18 primes tomato (*Solanum lycopersicum*) for enhanced drought stress responses. J. Plant Physiol..

[87] Schiff S., Tani C., Cimmino A., Mandala G., Cinelli T., Evidente A., Fiori M., Surico G., Marchi G. (2019). The colonization processes of *Myrtus communis* by strains of *Pseudomonas savastanoi* with a differential ability to produce phytohormones. Plant Pathol..

[88] Serdyuk O.P., Shirshikova G.N., Smolygina L.D., Butanaev A.M., Kreslavsky V.D., Yartseva N.V., Chekunova E.M. (2017). Agrobacterial *ipt* gene for cytokinin biosynthesis is found in phototrophic non-sulfur purple bacteria *Rhodobacter sphaeroides* and *Rhodopseudomonas palustris*. Russ. J. Genet..

[89] Zerrouk I.Z., Rahmoune B., Auer S., Roessler S., Lin T., Baluska F., Dobrev P.I., Motyka V., Ludwig-Mueller J. (2020). Growth and aluminum tolerance of maize roots mediated by auxin- and cytokinin-producing *Bacillus toyonensis* requires polar auxin transport. Environ. Exp. Bot..

[90] Chattopadhyay P., Banerjee G., Handique P.J. (2022). Use of an abscisic acid-producing *Bradyrhizobium japonicum* isolate as biocontrol agent against bacterial wilt disease caused by *Ralstonia solanacearum*. J. Plant Dis. Prot..

[91] Ding Z.-T., Zhang Z., Luo D., Zhou J.-Y., Zhong J., Yang J., Xiao L., Shu D., Tan H. (2015). Gene Overexpression and RNA Silencing Tools for the Genetic Manipulation of the S-(+)-Abscisic Acid Producing Ascomycete *Botrytis cinerea*. Int. J. Mol. Sci..

[92] Peeters K.J., Ameye M., Demeestere K., Audenaert K., Hofte M. (2020). Auxin, Abscisic Acid and Jasmonate Are the Central Players in Rice Sheath Rot Caused by *Sarocladium oryzae* and *Pseudomonas fuscovaginae*. Rice.

[93] Wang H., Wang S., He X., Xie M., Cai M., Zhu Y., Du S. (2023). A promising product: Abscisic acid-producing bacterial agents for restricting cadmium enrichment in field vegetable crops. Food Chem. X.

[94] North J.A., Narrowe A.B., Xiong W., Byerly K.M., Zhao G., Young S.J., Murali S., Wildenthal J.A., Cannon W.R., Wrighton K.C. (2020). A nitrogenase-like enzyme system catalyzes methionine, ethylene, and methane biogenesis. Science.

[95] Zhang M., Liu C., Xi D., Bi H., Cui Z., Zhuang Y., Yin H., Liu T. (2022). Metabolic Engineering of *Escherichia coli* for High-Level Production of Salicin. Acs Omega.

[96] Nguyen L.T.T., Park A.R., Van Le V., Hwang I., Kim J.-C. (2024). Exploration of a multifunctional biocontrol agent *Streptomyces* sp. JCK-8055 for the management of apple fire blight. Appl. Microbiol. Biotechnol..

[97] Reglinski T., Wurms K., Northcott G., Taylor J., Chee A.A., Parry F., Fehlmann C., Cooney J., Jensen D., Elmer P. (2024). Saccharin induces resistance against *Pseudomonas syringae* pv. *actinidiae* (Psa biovar 3) in glasshouse kiwifruit and orchard vines. Plant Pathol..

[98] Eng F., Haroth S., Feussner K., Meldau D., Rekhter D., Ischebeck T., Brodhun F., Feussner I. (2016). Optimized Jasmonic Acid Production by *Lasiodiplodia theobromae* Reveals Formation of Valuable Plant Secondary Metabolites. PLoS ONE.

[99] Eng Sanchez F., Gutierrez-Rojas M., Favela-Torres E. (2008). Studies on the effects of carbon:nitrogen ratio, inoculum type and yeast extract addition on jasmonic acid production by *Botryodiplodia theobromae* Pat. strain RC1. Rev. Iberoam. Micol..

[100] Go I.-H., Kim K.-J., Kim Y.-H. (2006). Optimal Conditions for the Production of (+)-Jasmonic acid by Diplodia gossypina ATCC10936. Korean J. Microbiol..

[101] Eng F., Marin J.E., Zienkiewicz K., Gutierrez-Rojas M., Favela-Torres E., Feussner I. (2021). Jasmonic acid biosynthesis by fungi: Derivatives, first evidence on biochemical pathways and culture conditions for production. Peerj.

[102] Veselova S., Nuzhnaya T., Maksimov I. (2024). The Role of Salicylic, Jasmonic Acid and Ethylene in the Development of the Resistance/Susceptibility of Wheat to the SnTox1-Producing Isolate of the Pathogenic Fungus *Stagonospora nodorum* (Berk.). Plants.

[103] Zhong Q., Xu Y., Rao Y. (2024). Mechanism of Rice Resistance to Bacterial Leaf Blight via Phytohormones. Plants.

[104] Yoshida M., Matsui Y., Ikarashi Y., Usui T., Osada H., Wakasugi H. (2007). Antiproliferating activity of the mitotic inhibitor pironetin against vindesine- and paclitaxel-resistant human small cell lung cancer H69 cells. Anticancer Res..

[105] Kobayashi S., Tsuchiya K., Harada T., Nishide M., Kurokawa T., Nakagawa T., Shimada N., Kobayashi K. (1994). Pironetin, a novel plant-growth regulator produced by *Streptomyces* sp. nk10958. 1. Taxonomy, production, isolation and preliminary characterization. J. Antibiot..

[106] Guesmi S., Mahjoubi M., Pujic P., Cherif A., Normand P., Sghaier H., Boubakri H. (2022). Biotechnological potential of *Kocuria rhizophila* PT10 isolated from roots of *Panicum turgidum*. Int. J. Environ. Sci. Technol..

[107] Goud M.S., Sharma S.K., Kharbikar L.L., Prasanna R., Sangwan S., Dahuja A., Dixit A. (2024). *Bacillus* species consortium with tryptophan-dependent and -independent pathways mediated production of IAA and its derivatives modulates soil biological properties, growth and yield of wheat. Plant Soil.

[108] Dellagi A., Quillere I., Hirel B. (2020). Beneficial soil-borne bacteria and fungi: A promising way to improve plant nitrogen acquisition. J. Exp. Bot..

[109] Khan A.L., Lee I.-J. (2013). Endophytic *Penicillium funiculosum* LHL06 secretes gibberellin that reprograms *Glycine max* L. growth during copper stress. BMC Plant Biol..

[110] Timofeeva A.M., Galyamova M.R., Sedykh S.E. (2024). How Do Plant Growth-Promoting Bacteria Use Plant Hormones to Regulate Stress Reactions?. Plants.

[111] Mughal N., Shoaib N., Chen J., Li Y., He Y., Fu M., Li X., He Y., Guo J., Deng J. (2024). Adaptive roles of cytokinins in enhancing plant resilience and yield against environmental stressors. Chemosphere.

[112] Romero-Muñoz M., Albacete A., Gálvez A., Piñero M.C., del Amor F.M., López-Marín J. (2022). The Use of Ecological Hydromulching Improves Growth in Escarole (*Cichorium endivia* L.) Plants Subjected to Drought Stress by Fine-Tuning Cytokinins and Abscisic Acid Balance. Agronomy.

[113] Zampieri E., Franchi E., Giovannini L., Brescia F., Sillo F., Fusini D., Pietrini I., Centritto M., Balestrini R. (2023). Diverse plant promoting bacterial species differentially improve tomato plant fitness under water stress. Front. Plant Sci..

[114] Li L., Feng Y., Qi F., Hao R. (2023). Research Progress of Piriformospora indica in Improving Plant Growth and Stress Resistance to Plant. J. Fungi.

[115] Pan Y., Liu B., Zhang W., Zhuang S., Wang H., Chen J., Xiao L., Li Y., Han D. (2024). Drought-induced assembly of rhizosphere mycobiomes shows beneficial effects on plant growth. Msystems.

[116] Ansari M.W., Trivedi D.K., Sahoo R.K., Gill S.S., Tuteja N. (2013). A critical review on fungi mediated plant responses with special emphasis to *Piriformospora indica* on improved production and protection of crops. Plant Physiol. Biochem..

[117] Zhang X., Wu F., Gu N., Yan X., Wang K., Dhanasekaran S., Gu X., Zhao L., Zhang H. (2020). Postharvest biological control of Rhizopus rot and the mechanisms involved in induced disease resistance of peaches by *Pichia membranefaciens*. Postharvest Biol. Technol..

[118] Edwards J.L. (2007). Enhancing the Ability of *Panicum virgatum* to Survive Flooding and Its Effects on Soil Activity When Used for Lakeshore Stabilization. Ph.D. Thesis.

[119] Ahmad F., Shah S.H., Jan A.S. (2023). Overexpression of the *DREB1A* gene under stress-inducible promoter delays leaf senescence and improves drought tolerance in rice. Cereal Res. Commun..

[120] Singh K., Gupta R., Shokat S., Iqbal N., Kocsy G., Perez-Perez J.M., Riyazuddin R. (2024). Ascorbate, plant hormones and their interactions during plant responses to biotic stress. Physiol. Plant..

[121] Browse J. (2009). Jasmonate Passes Muster: A Receptor and Targets for the Defense Hormone. Annu. Rev. Plant Biol..

[122] Okada K., Abe H., Arimura G.-i. (2015). Jasmonates Induce Both Defense Responses and Communication in Monocotyledonous and Dicotyledonous Plants. Plant Cell Physiol..

[123] He S., Huang K., Li B., Lu G., Wang A. (2023). Functional Analysis of a Salicylate Hydroxylase in *Sclerotinia sclerotiorum*. J. Fungi.

[124] Hao G., Naumann T.A., Vaughan M.M., McCormick S., Usgaard T., Kelly A., Ward T.J. (2019). Characterization of a *Fusarium graminearum* Salicylate Hydroxylase. Front. Microbiol..

[125] Liu L.X., Li W.N., Li X.L., Sun X.X., Yuan Q.P. (2019). Constructing an efficient salicylate biosynthesis platform by *Escherichia coli* chromosome integration. J. Biotechnol..

[126] Jimenez-Aleman G.H., Almeida-Trapp M., Fernández-Barbero G., Gimenez-Ibanez S., Reichelt M., Vadassery J., Mithöfer A., Caballero J., Boland W., Solano R. (2019). Omega hydroxylated JA-Ile is an endogenous bioactive jasmonate that signals through the canonical jasmonate signaling pathway. Biochim. Biophys. Acta-Mol. Cell Biol. Lipids.

[127] Guo D., Li J., Liu P., Wang Y., Cao N., Fang X., Wang T., Dong J. (2024). The jasmonate pathway promotes nodule symbiosis and suppresses host plant defense in *Medicago truncatula*. Mol. Plant.

[128] Miyamoto K., Shimizu T., Okada K. (2014). Transcriptional regulation of the biosynthesis of phytoalexin: A lesson from specialized metabolites in rice. Plant Biotechnol..

[129] Shimizu T., Lin F., Hasegawa M., Okada K., Nojiri H., Yamane H. (2012). Purification and Identification of Naringenin 7-*O*-Methyltransferase, a Key Enzyme in Biosynthesis of Flavonoid Phytoalexin Sakuranetin in Rice. J. Biol. Chem..

[130] Shimizu T., Miyamoto K., Miyamoto K., Minami E., Nishizawa Y., Iino M., Nojiri H., Yamane H., Okada K. (2013). OsJAR1 Contributes Mainly to Biosynthesis of the Stress-Induced Jasmonoyl-Isoleucine Involved in Defense Responses in Rice. Biosci. Biotechnol. Biochem..

[131] Kiryu M., Hamanaka M., Yoshitomi K., Mochizuki S., Akimitsu K., Gomi K. (2018). Rice *terpene synthase 18* (*OsTPS18*) encodes a sesquiterpene synthase that produces an antibacterial (*E*)-nerolidol against a bacterial pathogen of rice. J. Gen. Plant Pathol..

[132] Yoshitomi K., Taniguchi S., Tanaka K., Uji Y., Akimitsu K., Gomi K. (2016). Rice *terpene synthase 24* (*OsTPS24*) encodes a jasmonate-responsive monoterpene synthase that produces an antibacterial γ-terpinene against rice pathogen. J. Plant Physiol..

[133] Kiyama H., Matsunaga A., Suzuki G., Gomi K. (2021). Monoterpene geraniol produced by rice terpene synthase 21 suppresses the expression of cell-division related genes in the rice bacterial pathogen, *Xanthomonas oryzae* pv. oryzae. Physiol. Mol. Plant Pathol..

[134] Zhao Y., Song C., Brummell D.A., Qi S., Lin Q., Duan Y. (2021). Jasmonic acid treatment alleviates chilling injury in peach fruit by promoting sugar and ethylene metabolism. Food Chem..

[135] Kobayashi S., Tsuchiya K., Kurokawa T., Nakagawa T., Shimada N., Iitaka Y. (1994). Pironetin, a novel plant-growth regulator produced by *Streptomyces* sp. nk10958. 2. Structural elucidation. J. Antibiot..

[136] Altomare C., Pengue R., Favilla M., Evidente A., Visconti A. (2004). Structure-activity relationships of derivatives of fusapyrone, an antifungal metabolite of *Fusarium semitectum*. J. Agric. Food Chem..

[137] Kumagai T., Koyama Y., Oda K., Noda M., Matoba Y., Sugiyama M. (2010). Molecular Cloning and Heterologous Expression of a Biosynthetic Gene Cluster for the Antitubercular Agent D-Cycloserine Produced by *Streptomyces lavendulae*. Antimicrob. Agents Chemother..

[138] Choudhury A.R., Choi J., Walitang D.I., Trivedi P., Lee Y., Sa T. (2021). ACC deaminase and indole acetic acid producing endophytic bacterial co-inoculation improves physiological traits of red pepper (*Capsicum annum* L.) under salt stress. J. Plant Physiol..

[139] Hao J.-R., Li Y., Ge Y. (2024). Harnessing the plant microbiome for environmental sustainability: From ecological foundations to novel applications. Sci. Total Environ..

[140] Wang X., Chi Y., Song S. (2024). Important soil microbiota&apos;s effects on plants and soils: A comprehensive 30-year systematic literature review. Front. Microbiol..

[141] Chen X.H., Koumoutsi A., Scholz R., Eisenreich A., Schneider K., Heinemeyer I., Morgenstern B., Voss B., Hess W.R., Reva O. (2007). Comparative analysis of the complete genome sequence of the plant growth-promoting bacterium Bacillus amyloliquefaciens FZB42. Nat. Biotechnol..

[142] Bhattacharyya P.N., Jha D.K. (2012). Plant growth-promoting rhizobacteria (PGPR): Emergence in agriculture. World J. Microbiol. Biotechnol..

[143] Hider R.C., Kong X. (2010). Chemistry and biology of siderophores. Nat. Prod. Rep..

[144] Sorlin P., Brivet E., Jean-Pierre V., Aujoulat F., Besse A., Dupont C., Chiron R., Jumas-Bilak E., Menetrey Q., Marchandin H. (2024). Prevalence and variability of siderophore production in the *Achromobacter* genus. Microbiol. Spectr..

[145] Timofeeva A.M., Galyamova M.R., Sedykh S.E. (2022). Bacterial Siderophores: Classification, Biosynthesis, Perspectives of Use in Agriculture. Plants.

[146] Hu X., Boyer G.L. (1996). Siderophore-Mediated Aluminum Uptake by Bacillus megaterium ATCC 19213. Appl. Environ. Microbiol..

[147] Storey E.P., Boghozian R., Little J.L., Lowman D.W., Chakraborty R. (2006). Characterization of ‘Schizokinen’;: A dihydroxamate-type siderophore produced by *Rhizobium leguminosarum* IARI 917. Biometals.

[148] Fadeev E.A., Luo M., Groves J.T. (2005). Synthesis and structural modeling of the amphiphilic siderophore rhizobactin-1021 and its analogs. Bioorganic Med. Chem. Lett..

[149] De Vleesschauwer D., Djavaheri M., Bakker P.A.H.M., Hoefte M. (2008). *Pseudomonas fluorescens* WCS374r-Induced Systemic Resistance in Rice against *Magnaporthe oryzae* Is Based on Pseudobactin-Mediated Priming for a Salicylic Acid-Repressible Multifaceted Defense Response. Plant Physiol..

[150] Schalk I.J. (2025). Bacterial siderophores: Diversity, uptake pathways and applications. Nat. Rev. Microbiol..

[151] Feng Z., Sun H., Qin Y., Zhou Y., Zhu H., Yao Q. (2023). A synthetic community of siderophore-producing bacteria increases soil selenium bioavailability and plant uptake through regulation of the soil microbiome. Sci. Total Environ..

[152] Sun X., Zhang C., Bei S., Wang G., Geisen S., Bedoussac L., Christie P., Zhang J. (2022). High bacterial diversity and siderophore-producing bacteria collectively suppress *Fusarium oxysporum* in maize/faba bean intercropping. Front. Microbiol..

[153] Wang N., Wang T., Chen Y., Wang M., Lu Q., Wang K., Dou Z., Chi Z., Qiu W., Dai J. (2024). Microbiome convergence enables siderophore-secreting-rhizobacteria to improve iron nutrition and yield of peanut intercropped with maize. Nat. Commun..

[154] Getzke F., Hassani M.A., Cruesemann M., Malisic M., Zhang P., Ishigaki Y., Boehringer N., Fernandez A.J., Wang L., Ordon J. (2023). Cofunctioning of bacterial exometabolites drives root microbiota establishment. Proc. Natl. Acad. Sci. USA.

[155] Fliss O., Guay L.-D., Fliss I., Biron E. (2024). Synthesis and structure-activity study of the antimicrobial lipopeptide brevibacillin. RSC Med. Chem..

[156] Peng X., Wu H., Chen H.J., Zhang Y.J., Qiu D., Zhang Z.Y. (2019). Transcriptome profiling reveals candidate flavonol-related genes of Tetrastigma hemsleyanum under cold stress. BMC Genom..

[157] Jiang J., Gao L., Bie X., Lu Z., Liu H., Zhang C., Lu F., Zhao H. (2016). Identification of novel surfactin derivatives from NRPS modification of *Bacillus subtilis* and its antifungal activity against *Fusarium moniliforme*. BMC Microbiol..

[158] Modabber G., Sepahi A.A., Yazdian F., Rashedi H. (2023). Evaluation of production of lipopeptide biosurfactants and surfactin micelles by native *Bacillus* of Iran, for a broader application range. J. Surfactants Deterg..

[159] Cao C.-Y., Hou Z.-J., Ding M.-Z., Gao G.-R., Qiao B., Wei S.-Y., Cheng J.-S. (2024). Integrated Biofilm Modification and Transcriptional Analysis for Improving Fengycin Production in *Bacillus amyloliquefaciens*. Probiotics Antimicrob. Proteins.

[160] Fu R., Zhang H., Chang H., Zhang F., Chen W. (2020). Evaluation of Antifungal Mechanism of *Bacillus amyloliquefaciens* BA-16-8. Int. J. Agric. Biol..

[161] Kaki A.A., Smargiasso N., Ongena M., Ail M.K., Moula N., De Pauw E., Chaouche N.K. (2020). Characterization of New Fengycin Cyclic Lipopeptide Variants Produced by *Bacillus amyloliquefaciens* (ET) Originating from a Salt Lake of Eastern Algeria. Curr. Microbiol..

[162] Yaseen Y., Gancel F., Bechet M., Drider D., Jacques P. (2017). Study of the correlation between fengycin promoter expression and its production by *Bacillus subtilis* under different culture conditions and the impact on surfactin production. Arch. Microbiol..

[163] Jin P., Wang H., Tan Z., Xuan Z., Dahar G.Y., Li Q.X., Miao W., Liu W. (2020). Antifungal mechanism of bacillomycin D from *Bacillus velezensis* HN-2 against *Colletotrichum gloeosporioides* Penz. Pestic. Biochem. Physiol..

[164] Lin F., Zhu X., Sun J., Meng F., Lu Z., Lu Y. (2022). Bacillomycin D-C16 inhibits growth of *Fusarium verticillioides* and production of fumonisin B_1_ in maize kernels. Pestic. Biochem. Physiol..

[165] Qian S., Li J., Xu T., Diao E., Tang Y., Zhou X., Liu Y. (2023). Calcium lactate efficiently induces production of bacillomycin D in *Bacillus subtilis* NS-174 and inhibition of spore germination of *Aspergillus flavus* in rice. Int. J. Food Sci. Technol..

[166] Qian S., Lu H., Meng P., Zhang C., Lv F., Bie X., Lu Z. (2015). Effect of inulin on efficient production and regulatory biosynthesis of bacillomycin D in *Bacillus subtilis* fmbJ. Bioresour. Technol..

[167] Ebe S., Ohike T., Okanami M., Ano T. (2019). Components of rice husk biochar in promoting the growth, sporulation and iturin A production of *Bacillus* sp. strain IA. Z. Naturforschung Sect. C-A J. Biosci..

[168] Fujita S., Yokota K. (2019). Disease suppression by the cyclic lipopeptides iturin A and surfactin from *Bacillus* spp. against *Fusarium* wilt of lettuce. J. Gen. Plant Pathol..

[169] Klich M.A., Lax A.R., Bland J.M. (1991). Inhibition of some mycotoxigenic fungi by iturin A, a peptidolipid produced by Bacillus subtilis. Mycopathologia.

[170] Zhou S., Liu G., Zheng R., Sun C., Wu S. (2020). Structural and Functional Insights into Iturin W, a Novel Lipopeptide Produced by the Deep-Sea Bacterium *Bacillus* sp. Strain wsm-1. Appl. Environ. Microbiol..

[171] Chen N., Cai P., Zhang D., Zhang J., Zhong Z., Li Y.-X. (2024). Metabolic engineering of “last-line antibiotic” colistin in Paenibacillus polymyxa. Metab. Eng..

[172] Hossain A., Ali M.A., Lin L., Luo J., You Y., Masum M.M.I., Jiang Y., Wang Y., Li B., An Q. (2023). Biocontrol of Soft Rot *Dickeya* and *Pectobacterium* Pathogens by Broad-Spectrum Antagonistic Bacteria within *Paenibacillus polymyxa* Complex. Microorganisms.

[173] Shi Q., Zhang J., Fu Q., Hao G., Liang C., Duan F., Zhao H., Song W. (2024). Biocontrol Efficacy and Induced Resistance of *Paenibacillus polymyxa* J2-4 Against *Meloidogyne incognita* Infection in Cucumber. Phytopathology.

[174] Wang H., Wang N., Tan Y., Mi Q., Mao Y., Zhao C., Tian X., Liu W., Huang L. (2023). *Paenibacillus polymyxa* YLC1: A promising antagonistic strain for biocontrol of *Pseudomonas syringae* pv. *actinidiae*, causing kiwifruit bacterial canker. Pest Manag. Sci..

[175] Yang M., Song Y., Ma H., Li Z., Ding J., Yin T., Niu K., Sun S., Qi J., Lu G. (2024). Unveiling the hidden world: How arbuscular mycorrhizal fungi and its regulated core fungi modify the composition and metabolism of soybean rhizosphere microbiome. Environ. Microbiome.

[176] Li D., Wang Y., Chen C., Zeng M., Li Q., Jia Q., Liu X., Hou Y., Fan C., Chen Y. (2022). Advances in several important antimicrobial lipopeptids from *Bacillus* spp.. Sheng Wu Gong Cheng Xue Bao = Chin. J. Biotechnol..

[177] Li G., Liu B., Shang Y., Yu Z., Zhang R. (2012). Novel activity evaluation and subsequent partial purification of antimicrobial peptides produced by *Bacillus subtilis* LFB112. Ann. Microbiol..

[178] Girdhar M., Sen A., Nigam A., Oswalia J., Kumar S., Gupta R. (2024). Antimicrobial peptide-based strategies to overcome antimicrobial resistance. Arch. Microbiol..

[179] Ajuna H.B., Lim H.-I., Moon J.-H., Won S.-J., Choub V., Choi S.-I., Yun J.-Y., Ahn Y.S. (2024). The prospect of antimicrobial peptides from *Bacillus* species with biological control potential against insect pests and diseases of economic importance in agriculture, forestry and fruit tree production. Biotechnol. Biotechnol. Equip..

[180] Assena M.W., Pfannstiel J., Rasche F. (2024). Inhibitory activity of bacterial lipopeptides against *Fusarium oxysporum* f.sp. Strigae. BMC Microbiol..

[181] Jemil N., Besbes I., Gharbi Y., Triki M.A., Cheffi M., Manresa A., Nasri M., Hmidet N. (2024). *Bacillus methylotrophicus* DCS1: Production of Different Lipopeptide Families, In Vitro Antifungal Activity and Suppression of Fusarium Wilt in Tomato Plants. Curr. Microbiol..

[182] Lv Z., Li R., Shi C., Lu Z., Meng F., Bie X. (2024). The efficient synthesis strategy of bacillomycin D in *Bacillus* amyloliquefaciens fmbJ. Process Biochem..

[183] Miljakovic D., Marinkovic J., Tamindzic G., Milosevic D., Ignjatov M., Karacic V., Jaksic S. (2024). Bio-Priming with *Bacillus* Isolates Suppresses Seed Infection and Improves the Germination of Garden Peas in the Presence of *Fusarium* Strains. J. Fungi.

[184] Yang D., Zhang X., Li Z., Chu R., Shah S., Wang X., Zhang X. (2024). Antagonistic effect of *Bacillus* and *Pseudomonas* combinations against *Fusarium oxysporum* and their effect on disease resistance and growth promotion in watermelon. J. Appl. Microbiol..

[185] Yang R., Ye W., Liu P., Li J., Lu M., Wang Z., Shao D. (2024). Endophytic Bacillus amyloliquefaciens Mdgb15 is a potential biocontrol agent against tree peony gray mold caused by Botrytis cinerea. Eur. J. Plant Pathol..

[186] Chen Y.W., Liu X.C., Lv F.X., Li P. (2019). Characterization of three regulatory genes involved in enduracidin biosynthesis and improvement of enduracidin production in *Streptomyces fungicidicus*. J. Appl. Microbiol..

[187] Liu L., Hu W., Li W.-j., Wang S.-y., Lu D., Tian X.-j., Mao Y.-q., Liu J., Chen J.-h. (2019). Heavy-ion mutagenesis significantly enhances enduracidin production by *Streptomyces fungicidicus*. Eng. Life Sci..

[188] Liu L., Li W.-j., Hu W., Pan X.-h., Tian X.-j., Mao Y.-q., Chen J.-h. (2019). Assessment of enduracidin production from sweet sorghum juice by *Streptomyces fungicidicus* M30. Ind. Crops Prod..

[189] Zhang J., He Z., Xu J., Song S., Zhu Q., Wu G., Guan Y., Wu X., Yue R., Wang Y. (2020). Semi-rational mutagenesis of an industrial *Streptomyces fungicidicus* strain for improved enduracidin productivity. Appl. Microbiol. Biotechnol..

[190] Chen J.-S., Su M., Shao L., Wang Y.-X., Lin H.-M., Chen D.-J. (2016). Investigation of halogenation during the biosynthesis of ramoplanin in *Actinoplanes* sp ATCC33076. Appl. Microbiol. Biotechnol..

[191] Du X., Li Y., Zhou Q., Xu Y. (2015). Regulation of gene expression in *Pseudomonas aeruginosa* M18 by phenazine-1-carboxylic acid. Appl. Microbiol. Biotechnol..

[192] Wang G., Huang X., Li S., Huang J., Wei X., Li Y., Xu Y. (2012). The RNA Chaperone Hfq Regulates Antibiotic Biosynthesis in the Rhizobacterium *Pseudomonas aeruginosa* M18. J. Bacteriol..

[193] Wei X., Huang X., Tang L., Wu D., Xu Y. (2013). Global Control of GacA in Secondary Metabolism, Primary Metabolism, Secretion Systems, and Motility in the Rhizobacterium *Pseudomonas aeruginosa* M18. J. Bacteriol..

[194] Dong D., Li M., Zhang T., Niu Z., Xue G., Bai H., Zhao W., Yu J., Jiang W., Wu H. (2023). Antagonistic Activity of *Streptomyces alfalfae* 11F against *Fusarium* Wilt of Watermelon and Transcriptome Analysis Provides Insights into the Synthesis of Phenazine-1-Carboxamide. Plants.

[195] Li L., Ran T., Zhu H., Yin M., Yu W., Zou J., Li L., Ye Y., Sun H., Wang W. (2024). Molecular Mechanism of Fusarium Fungus Inhibition by Phenazine-1-carboxamide. J. Agric. Food Chem..

[196] Morohoshi T., Yabe N., Yaguchi N., Xie X., Someya N. (2022). Regulation of phenazine-1-carboxamide production by quorum sensing in type strains of *Pseudomonas chlororaphis* subsp. chlororaphis and Pseudomonas chlororaphis subsp. piscium. J. Biosci. Bioeng..

[197] Qi Z., Liu F., Li D., Yin J., Wang D., Ahmed N., Ma Y., Zhou J.-J., Chen Z. (2024). Phenazine-1-carboxamide Regulates Pyruvate Dehydrogenase of Phytopathogenic Fungi to Control Tea Leaf Spot Caused by *Didymella segeticola*. Phytopathology.

[198] Shahid I., Rizwan M., Baig D.N., Saleem R.S., Malik K.A., Mehnaz S. (2017). Secondary Metabolites Production and Plant Growth Promotion by *Pseudomonas chlororaphis* and *P*. *aurantiaca* Strains Isolated from Cactus, Cotton, and Para Grass. J. Microbiol. Biotechnol..

[199] Sokolowski W., Marek-Kozaczuk M., Sosnowski P., Sajnaga E., Jach M.E., Karas M.A. (2024). Profiling Metabolites with Antifungal Activities from Endophytic Plant-Beneficial Strains of *Pseudomonas chlororaphis* Isolated from *Chamaecytisus albus* (Hack.) Rothm. Molecules.

[200] Yu J.M., Wang D., Pierson L.S., Pierson E.A. (2018). Effect of Producing Different Phenazines on Bacterial Fitness and Biological Control in *Pseudomonas chlororaphis* 30-84. Plant Pathol. J..

[201] Bignell D.R.D., Fyans J.K., Cheng Z. (2014). Phytotoxins produced by plant pathogenic *Streptomyces* species. J. Appl. Microbiol..

[202] Li X., Yan Y., Xie S., Li Z., Xia H. (2023). Enhancement of milbemycins production by phosphopantetheinyl transferase and regulatory pathway engineering in *Streptomyces bingchenggensis*. World J. Microbiol. Biotechnol..

[203] Yan Y.-S., Xia H.-Y. (2021). Recent advances in the research of milbemycin biosynthesis and regulation as well as strategies for strain improvement. Arch. Microbiol..

[204] Yan Y.-S., Zou L.-S., Wei H.-G., Yang M.-Y., Yang Y.-Q., Li X.-F., Xia H.-Y. (2024). An atypical two-component system, AtcR/AtcK, simultaneously regulates the biosynthesis of multiple secondary metabolites in *Streptomyces bingchenggensis*. Appl. Environ. Microbiol..

[205] Yan Y.-S., Yang Y.-Q., Zhou L.-S., Zhang L., Xia H.-Y. (2022). MilR3, a unique SARP family pleiotropic regulator in *Streptomyces bingchenggensis*. Arch. Microbiol..

[206] Nie C., Huang X., Xiang T., Wang Z., Zhang X. (2024). Discovery and characterization of the PpqI/R quorum sensing system activated by GacS/A and Hfq in *Pseudomonas protegens* H78. Microbiol. Res..

[207] Wang Z., Huang X., Liu Y., Yang G., Liu Y., Zhang X. (2017). GacS/GacA activates pyoluteorin biosynthesis through Gac/Rsm-RsmE cascade and RsmA/RsmE-driven feedback loop in *Pseudomonas protegens* H78. Mol. Microbiol..

[208] Chenniappan C., Narayanasamy M., Daniel G.M., Ramaraj G.B., Ponnusamy P., Sekar J., Ramalingam P.V. (2019). Biocontrol efficiency of native plant growth promoting rhizobacteria against rhizome rot disease of turmeric. Biol. Control.

[209] Sun M.M., Xing F.Y., Pan S., Di J.F., Zeng S., Liu J. (2013). Low-dose anisomycin is sufficient to alter the bio-behaviors of Jurkat T cells. Cent. Eur. J. Biol..

[210] Zhang S., Liu Q., Han Y., Han J., Yan Z., Wang Y., Zhang X. (2019). Nematophin, an Antimicrobial Dipeptide Compound From *Xenorhabdus nematophila* YL001 as a Potent Biopesticide for *Rhizoctonia solani* Control. Front. Microbiol..

[211] Dreyer J., Rautenbach M., Booysen E., van Staden A.D., Deane S.M., Dicks L.M.T. (2019). *Xenorhabdus khoisanae* SB10 produces Lys-rich PAX lipopeptides and a Xenocoumacin in its antimicrobial complex. BMC Microbiol..

[212] Dong Y., Li X., Duan J., Qin Y., Yang X., Ren J., Li G. (2020). Improving the Yield of Xenocoumacin 1 Enabled by In Situ Product Removal. ACS Omega.

[213] Cui J., Zhang X. (2021). Development and high yield strategies of microbial-derived antibiotics in agriculture. Sheng Wu Gong Cheng Xue Bao = Chin. J. Biotechnol..

[214] Pan X., Cai J. (2024). Comparative transcriptome analysis of doramectin-producing *Streptomyces avermitilis* N72 and its mutant strains. World J. Microbiol. Biotechnol..

[215] Cerna-Chavez E., Rodriguez-Rodriguez J.F., Garcia-Conde K.B., Ochoa-Fuentes Y.M. (2024). Potential of *Streptomyces avermitilis*: A Review on Avermectin Production and Its Biocidal Effect. Metabolites.

[216] Vaishnav A., Kumari S., Jain S., Varma A., Choudhary D.K. (2015). Putative bacterial volatile-mediated growth in soybean (*Glycine max* L. Merrill) and expression of induced proteins under salt stress. J. Appl. Microbiol..

[217] Zaid D.S., Li W., Yang S., Li Y. (2023). Identification of bioactive compounds of *Bacillus velezensis* HNA3 that contribute to its dual effects as plant growth promoter and biocontrol against post-harvested fungi. Microbiol. Spectr..

[218] Yasmin H., Shah Z.A., Mumtaz S., Ilyas N., Rashid U., Alsahli A.A., Chung Y.S. (2023). Alleviation of banded leaf and sheath blight disease incidence in maize by bacterial volatile organic compounds and molecular docking of targeted inhibitors in Rhizoctonia solani. Front. Plant Sci..

[219] Luo L., Zhao C., Wang E., Raza A., Yin C. (2022). *Bacillus amyloliquefaciens* as an excellent agent for biofertilizer and biocontrol in agriculture: An overview for its mechanisms. Microbiol. Res..

[220] Hashem A., Tabassum B., Abd Allah E.F. (2019). *Bacillus subtilis*: A plant-growth promoting rhizobacterium that also impacts biotic stress. Saudi J. Biol. Sci..

[221] Zhou J.-Y., Li X., Zheng J.-Y., Dai C.-C. (2016). Volatiles released by endophytic *Pseudomonas fluorescens* promoting the growth and volatile oil accumulation in *Atractylodes lancea*. Plant Physiol. Biochem..

[222] Law J.W.-F., Ser H.-L., Khan T.M., Chuah L.-H., Pusparajah P., Chan K.-G., Goh B.-H., Lee L.-H. (2017). The Potential of *Streptomyces* as Biocontrol Agents against the Rice Blast Fungus, *Magnaporthe oryzae* (*Pyricularia oryzae*). Front. Microbiol..

[223] Li Q., Jiang Y., Ning P., Zheng L., Huang J., Li G., Jiang D., Hsiang T. (2011). Suppression of *Magnaporthe oryzae* by culture filtrates of *Streptomyces globisporus* JK-1. Biol. Control.

[224] Li Q., Ning P., Zheng L., Huang J., Li G., Hsiang T. (2012). Effects of volatile substances of *Streptomyces globisporus* JK-1 on control of *Botrytis cinerea* on tomato fruit. Biol. Control.

[225] Li Q., Ning P., Zheng L., Huang J., Li G., Hsiang T. (2010). Fumigant activity of volatiles of *Streptomyces globisporus* JK-1 against *Penicillium italicum* on *Citrus microcarpa*. Postharvest Biol. Technol..

[226] Lyu A., Liu H., Che H., Yang L., Zhang J., Wu M., Chen W., Li G. (2017). Reveromycins A and B from Streptomyces sp 3-10: Antifungal Activity against Plant Pathogenic Fungi In vitro and in a Strawberry Food Model System. Front. Microbiol..

[227] Lyu A., Yang L., Wu M., Zhang J., Li G. (2020). High Efficacy of the Volatile Organic Compounds of *Streptomyces yanglinensis* 3-10 in Suppression of *Aspergillus* Contamination on Peanut Kernels. Front. Microbiol..

[228] Shakeel Q., Lyu A., Zhang J., Wu M., Li G., Hsiang T., Yang L. (2018). Biocontrol of *Aspergillus flavus* on Peanut Kernels Using *Streptomyces yansingensis* 3-10. Front. Microbiol..

[229] Boukaew S., Prasertsan P. (2020). Efficacy of volatile compounds from *Streptomyces philanthi* RL-1-178 as a biofumigant for controlling growth and aflatoxin production of the two aflatoxin-producing fungi on stored soybean seeds. J. Appl. Microbiol..

[230] Boukaew S., Prasertsan P., Petlamul W., Cheirsilp B. (2024). Palm oil decanter cake wastes as alternative nutrient sources for production of enzymes from *Streptomyces philanthi* RM-1-138 and the efficacy of its culture filtrate as an antimicrobial agent against plant pathogenic fungi and bacteria. Biomass Convers. Biorefin..

[231] Boukaew S., Petlamul W., Bunkrongcheap R., Chookaew T., Kabbua T., Thippated A., Prasertsan P. (2018). Fumigant activity of volatile compounds of *Streptomyces philanthi* RM-1-138 and pure chemicals (acetophenone and phenylethyl alcohol) against anthracnose pathogen in postharvest chili fruit. Crop Prot..

[232] Boukaew S., Prasertsan P., Troulet C., Bardin M. (2017). Biological control of tomato gray mold caused by *Botrytis cinerea* by using *Streptomyces* spp.. Biocontrol.

[233] Liu C.X., Chen L.S., He Z.L., Zhang Z., Xu Y.M., Li Z.G., Peng Y.H., Deng N., Chen Y.Z. (2021). Integration and Potential Application Ability of Culturable Functional Microorganism in Oil Tea Camellia. Indian J. Microbiol..

[234] Cortes-Solis Y., Tovar-Rocha V., Tovar-Rocha J.C., Santoyo G., Rocha-Granados M.d.C. (2023). Growth parameters of blueberry (*Vaccinium* spp.) plants inoculated with *Pseudomonas fluorescens*. Acta Biol. Colomb..

[235] Hernandez-Leon R., Rojas-Solis D., Contreras-Perez M., del Carmen Orozco-Mosqueda M., Macias-Rodriguez L.I., Reyes-de la Cruz H., Valencia-Cantero E., Santoyo G. (2015). Characterization of the antifungal and plant growth-promoting effects of diffusible and volatile organic compounds produced by *Pseudomonas fluorescens* strains. Biol. Control.

[236] Rocha-Granados M.D.C., Cubillo-Constantino M.A., Delgado-Valerio P., GarcÍA-MagaÑA J., Santoyo G. (2019). Aumento de tolerancia de Casuarina equisetifolia a cloruro de sodio mediado por *Pseudomonas fluorescens*. Biotecnol. Sect. Agropecu. Agroind..

[237] Rojas-Solís D., Hernández-Pacheco C.E., Santoyo G. (2016). Evaluation of *Bacillus* and *Pseudomonas* to colonize the rhizosphere and their effect on growth promotion in tomato (*Physalis ixocarpa* Brot. ex Horm.). Rev. Chapingo. Ser. Hortic..

[238] Gong A.-D., Dong F.-Y., Hu M.-J., Kong X.-W., Wei F.-F., Gong S.-J., Zhang Y.-M., Zhang J.-B., Wu A.-B., Liao Y.-C. (2019). Antifungal activity of volatile emitted from *Enterobacter asburiae* Vt-7 against *Aspergillus flavus* and aflatoxins in peanuts during storage. Food Control.

[239] Alijani Z., Amini J., Ashengroph M., Bahramnejad B. (2019). Antifungal activity of volatile compounds produced by *Staphylococcus sciuri* strain MarR44 and its potential for the biocontrol of *Colletotrichum nymphaeae*, causal agent strawberry anthracnose. Int. J. Food Microbiol..

[240] Pena L.C., Jungklaus G.H., Savi D.C., Ferreira-Maba L., Servienski A., Maia B.H.L.N.S., Annies V., Galli-Terasawa L.V., Glienke C., Kava V. (2019). *Muscodor brasiliensis* sp. nov. produces volatile organic compounds with activity against *Penicillium digitatum*. Microbiol. Res..

[241] Siri-udom S., Suwannarach N., Lumyong S. (2016). Existence of *Muscodor vitigenus*, *M-equiseti* and *M-heveae* sp nov in leaves of the rubber tree (*Hevea brasiliensis* Mull.Arg.), and their biocontrol potential. Ann. Microbiol..

[242] Rajulu M.B.G., Suryanarayanan T.S., Murali T.S., Thirunavukkarasu N., Venkatesan G. (2021). Minor species of foliar fungal endophyte communities: Do they matter?. Mycol. Prog..

[243] Soto C. F., Tramón P. C., Aqueveque M. P., de Bruijn J. (2018). Microorganismos antagonistas que inhiben el desarrollo de patógenos en post-cosecha de limones (*Citrus limon* L.). Chil. J. Agric. Anim. Sci..

[244] Suwannarach N., Kumla J., Bussaban B., Nuangmek W., Matsui K., Lumyong S. (2013). Biofumigation with the endophytic fungus *Nodulisporium* spp. CMU-UPE34 to control postharvest decay of citrus fruit. Crop Prot..

[245] Aplin J.J. (2019). Use of Non-Saccharomyces Yeasts for Reducing the Ethanol Contents of Red Wine. Ph.D. Thesis.

[246] Contarino R., Brighina S., Fallico B., Cirvilleri G., Parafati L., Restuccia C. (2019). Volatile organic compounds (VOCs) produced by biocontrol yeasts. Food Microbiol..

[247] Oro L., Feliziani E., Ciani M., Romanazzi G., Comitini F. (2018). Volatile organic compounds from *Wickerhamomyces anomalus*, *Metschnikowia pulcherrima* and *Saccharomyces cerevisiae* inhibit growth of decay causing fungi and control postharvest diseases of strawberries. Int. J. Food Microbiol..

[248] Orozco-Mosqueda M.D.C., Kumar A., Fadiji A.E., Babalola O.O., Puopolo G., Santoyo G. (2023). Agroecological Management of the Grey Mould Fungus *Botrytis cinerea* by Plant Growth-Promoting Bacteria. Plants.

[249] Wang X., Glawe D.A., Kramer E., Weller D., Okubara P.A. (2018). Biological Control of *Botrytis cinerea*: Interactions with Native Vineyard Yeasts from Washington State. Phytopathology.

[250] Huang R., Li G.Q., Zhang J., Yang L., Che H.J., Jiang D.H., Huang H.C. (2011). Control of Postharvest Botrytis Fruit Rot of Strawberry by Volatile Organic Compounds of *Candida intermedia*. Phytopathology.

[251] Tenea G.N., Cajas B.A., Sanchez B.C. (2023). Inhibitory-like Substances Produced by Yeasts Isolated from Andean Blueberries: Prospective Food Antimicrobials. Foods.

[252] Costa O.Y.A., Raaijmakers J.M., Kuramae E.E. (2018). Microbial Extracellular Polymeric Substances: Ecological Function and Impact on Soil Aggregation. Front. Microbiol..

[253] Sandhya V., Ali S.Z. (2015). The production of exopolysaccharide by *Pseudomonas putida* GAP-P45 under various abiotic stress conditions and its role in soil aggregation. Microbiology.

[254] Azizi A., Gilandeh Y.A., Mesri-Gundoshmian T., Saleh-Bigdeli A.A., Moghaddam H.A. (2020). Classification of soil aggregates: A novel approach based on deep learning. Soil Tillage Res..

[255] Netrusov A.I., Liyaskina E.V., Kurgaeva I.V., Liyaskina A.U., Yang G., Revin V.V. (2023). Exopolysaccharides Producing Bacteria: A Review. Microorganisms.

[256] Naseem H., Ahsan M., Shahid M.A., Khan N. (2018). Exopolysaccharides producing rhizobacteria and their role in plant growth and drought tolerance. J. Basic Microbiol..

[257] Liu X.L., Ji B., Li A.J. (2023). Enhancing biolipid production and self-flocculation of *Chlorella vulgaris* by extracellular polymeric substances from granular sludge with CO_2_ addition: Microscopic mechanism of microalgae-bacteria symbiosis. Water Res..

[258] Flemming H.-C., Wingender J., Szewzyk U., Steinberg P., Rice S.A., Kjelleberg S. (2016). Biofilms: An emergent form of bacterial life. Nat. Rev. Microbiol..

[259] Vardharajula S., Ali S.Z., Grover M., Reddy G., Bandi V. (2011). Drought-tolerant plant growth promoting *Bacillus* spp.: Effect on growth, osmolytes, and antioxidant status of maize under drought stress. J. Plant Interact..

[260] Yin Z.-y., Yuan Y.-c., Zhang R., Gan J.-t., Yu L., Qiu X.-h., Chen R.-p., Wang Q. (2024). Understanding *Bacillus* response to salt stress: Growth inhibition, enhanced EPS secretion, and molecular adaptation mechanisms. Process Biochem..

[261] Rizzo M.G., Zammuto V., Spano A., Gugliandolo C., Calabrese G., Guglielmino S. (2024). Anti-inflammatory effects in LPS-induced macrophages and antibiofilm activity of the mannose-rich exopolysaccharide produced by Bacillus licheniformis B3-15. Heliyon.

[262] More T.T., Yadav J.S.S., Yan S., Tyagi R.D., Surampalli R.Y. (2014). Extracellular polymeric substances of bacteria and their potential environmental applications. J. Environ. Manag..

[263] Ahmad I., Akhtar M.J., Asghar H.N., Ghafoor U., Shahid M. (2016). Differential Effects of Plant Growth-Promoting Rhizobacteria on Maize Growth and Cadmium Uptake. J. Plant Growth Regul..

[264] Esmaeel Q., Pupin M., Jacques P., Leclere V. (2018). Nonribosomal peptides and polyketides of *Burkholderia*: New compounds potentially implicated in biocontrol and pharmaceuticals. Environ. Sci. Pollut. Res..

[265] Dimkic I., Janakiev T., Petrovic M., Degrassi G., Fira D. (2022). Plant-associated Bacillus and Pseudomonas antimicrobial activities in plant disease suppression via biological control mechanisms—A review. Physiol. Mol. Plant Pathol..

[266] Souto A.L., Sylvestre M., Tolke E.D., Tavares J.F., Barbosa-Filho J.M., Cebrian-Torrejon G. (2021). Plant-Derived Pesticides as an Alternative to Pest Management and Sustainable Agricultural Production: Prospects, Applications and Challenges. Molecules.

[267] Yang W.Q., Chen X., Li Y.L., Guo S.F., Wang Z., Yu X.L. (2020). Advances in Pharmacological Activities of Terpenoids. Nat. Prod. Commun..

[268] Paddon C.J., Westfall P.J., Pitera D.J., Benjamin K., Fisher K., McPhee D., Leavell M.D., Tai A., Main A., Eng D. (2013). High-level semi-synthetic production of the potent antimalarial artemisinin. Nature.

[269] Tae H., Sohng J.K., Park K. (2009). MapsiDB: An integrated web database for type I polyketide synthases. Bioprocess Biosyst. Eng..

[270] Tae H., Kong E.-B., Park K. (2007). ASMPKS: An analysis system for modular polyketide synthases. BMC Bioinform..

[271] Hoeger P.H. (2004). Antimicrobial susceptibility of skin-colonizing *S-aureus* strains in children with atopic dermatitis. Pediatr. Allergy Immunol..

[272] Geris R., Simpson T.J. (2009). Meroterpenoids produced by fungi. Nat. Prod. Rep..

[273] Chen J.-X., Xia D.-D., Yang X.-Q., Yang Y.-B., Ding Z.-T. (2022). The antifeedant and antifungal cryptic metabolites isolated from tobacco endophytes induced by host medium and coculture. Fitoterapia.

[274] Li C., Shao Y., Li W., Yin T., Li H., Yan H., Guo X., Liu B., He B. (2022). Hybrid Diterpenic Meroterpenoids from an Endophytic *Penicillium* sp. Induced by Chemical Epigenetic Manipulation. J. Nat. Prod..

[275] Kumar A., Das A., Singh D., Das M.K., Srivastava G.P., Singh J.P., Tilgam J., Thapa S., Das S., Chakdar H. (2023). Soil health restoration in degraded lands: A microbiological perspective. Land Degrad. Dev..

[276] Sritongon N., Boonlue S., Mongkolthanaruk W., Jogloy S., Riddech N. (2023). The combination of multiple plant growth promotion and hydrolytic enzyme producing rhizobacteria and their effect on Jerusalem artichoke growth improvement. Sci. Rep..

[277] Fanai A., Bohia B., Lalremruati F., Lalhriatpuii N., Lalrokimi, Lalmuanpuii R., Singh P.K., Zothanpuia. (2024). Plant growth promoting bacteria (PGPB)-induced plant adaptations to stresses: An updated review. Peerj.

[278] Weidner S., Koller R., Latz E., Kowalchuk G., Bonkowski M., Scheu S., Jousset A. (2015). Bacterial diversity amplifies nutrient-based plant-soil feedbacks. Funct. Ecol..

[279] Agarwal P., Giri B.S., Rani R. (2020). Unravelling the Role of Rhizospheric Plant-Microbe Synergy in Phytore-mediation: A Genomic Perspective. Curr. Genom..

[280] Aasfar A., Bargaz A., Yaakoubi K., Hilali A., Bennis I., Zeroual Y., Kadmiri I.M. (2021). Nitrogen Fixing *Azotobacter* Species as Potential Soil Biological Enhancers for Crop Nutrition and Yield Stability. Front. Microbiol..

[281] Rabari A., Ruparelia J., Jha C.K., Sayyed R.Z., Mitra D., Priyadarshini A., Senapati A., Panneerselvam P., Das Mohapatra P.K. (2023). Articulating beneficial rhizobacteria-mediated plant defenses through induced systemic resistance: A review. Pedosphere.

[282] Walters D.R., Fountaine J.M. (2009). Practical application of induced resistance to plant diseases: An appraisal of effectiveness under field conditions. J. Agric. Sci..

[283] Pieterse C.M., Zamioudis C., Berendsen R.L., Weller D.M., Van Wees S.C., Bakker P.A. (2014). Induced Systemic Resistance by Beneficial Microbes. Annu. Rev. Phytopathol..

[284] Yu Y., Gui Y., Li Z., Jiang C., Guo J., Niu D. (2022). Induced Systemic Resistance for Improving Plant Immunity by Beneficial Microbes. Plants.

[285] van Oosten V. (2007). Induced Pathogen and Insect Resistance in Arabidopsis: Transcriptomics and Specificity of Defense. Ph.D. Thesis.

[286] Sahu P.K., Jayalakshmi K., Tilgam J., Gupta A., Nagaraju Y., Kumar A., Hamid S., Singh H.V., Minkina T., Rajput V.D. (2022). ROS generated from biotic stress: Effects on plants and alleviation by endophytic microbes. Front. Plant Sci..

[287] Nie P., Li X., Wang S., Guo J., Zhao H., Niu D. (2017). Induced Systemic Resistance against *Botrytis cinerea* by *Bacillus cereus* AR156 through a JA/ET- and NPR1-Dependent Signaling Pathway and Activates PAMP-Triggered Immunity in *Arabidopsis*. Front. Plant Sci..

[288] Vlot A.C., Sales J.H., Lenk M., Bauer K., Brambilla A., Sommer A., Chen Y., Wenig M., Nayem S. (2021). Systemic propagation of immunity in plants. New Phytol..

[289] Wang D., Luo W.Z., Zhang D.D., Li R., Kong Z.Q., Song J., Dai X.F., Alkan N., Chen J.Y. (2023). Insights into the biocontrol function of a burkholderia gladioli strain against botrytis cinerea. Microbiology Spectrum..

[290] Karacic V., Miljakovic D., Marinkovic J., Ignjatov M., Milosevic D., Tamindzic G., Ivanovic M. (2024). *Bacillus* Species: Excellent Biocontrol Agents against Tomato Diseases. Microorganisms.

[291] Janaagal M., Sharma P., Kumari G., Gulia H., Suresh G., Tallapragada S., Devi S., Lakra N., Arya S.S., Pooja P. (2024). Revolutionizing High Temperature Stress Relief: Exploring the Latest Advances in Salicylic Acid Application. J. Crop Health.

[292] Duan S., Feng G., Limpens E., Bonfante P., Xie X., Zhang L. (2024). Cross-kingdom nutrient exchange in the plant-arbuscular mycorrhizal fungus-bacterium continuum. Nat. Rev. Microbiol..

[293] Sun W., Shahrajabian M.H. (2023). The Application of Arbuscular Mycorrhizal Fungi as Microbial Biostimulant, Sustainable Approaches in Modern Agriculture. Plants.

[294] Wahab A., Muhammad M., Munir A., Abdi G., Zaman W., Ayaz A., Khizar C., Reddy S.P.P. (2023). Role of Arbuscular Mycorrhizal Fungi in Regulating Growth, Enhancing Productivity, and Potentially Influencing Ecosystems under Abiotic and Biotic Stresses. Plants.

[295] Ahlawat O.P., Yadav D., Walia N., Kashyap P.L., Sharma P., Tiwari R. (2024). Root Exudates and Their Significance in Abiotic Stress Amelioration in Plants: A Review. J. Plant Growth Regul..

[296] Chen L., Gong J., Jin J., Wang L., Chen X., Wang C., Tang M., Liu J., Wen Z., Yang Y. (2024). Intrinsic and extrinsic regulatory mechanisms of *Pseudomonas palleroniana* GZNU148 for enhancing *Themeda japonica* tolerance to drought stress. Plant Soil.

[297] Munir N., Hanif M., Abideen Z., Sohail M., El-Keblawy A., Radicetti E., Mancinelli R., Haider G. (2022). Mechanisms and Strategies of Plant Microbiome Interactions to Mitigate Abiotic Stresses. Agronomy.

[298] Vishwakarma K., Kumar N., Shandilya C., Mohapatra S., Bhayana S., Varma A. (2020). Revisiting Plant-Microbe Interactions and Microbial Consortia Application for Enhancing Sustainable Agriculture: A Review. Front. Microbiol..

[299] Korenblum E., Massalha H., Aharoni A. (2022). Plant-microbe interactions in the rhizosphere via a circular metabolic economy. Plant Cell.

[300] Malik D.K., Sindhu S.S. (2008). Transposon-derived mutants of Pseudomonas strains altered in indole acetic acid production: Effect on nodulation and plant growth in green gram (*Vigna radiata* L.). Physiol. Mol. Biol. Plants Int. J. Funct. Plant Biol..

[301] Poprzen T., Nikolic I., Krstic-Milosevic D., Uzelac B., Trifunovic-Momcilov M., Markovic M., Radulovic O. (2023). Characterization of the IAA-Producing and -Degrading *Pseudomonas* Strains Regulating Growth of the Common Duckweed (*Lemna minor* L.). Int. J. Mol. Sci..

[302] Bhupenchandra I., Chongtham S.K., Devi A.G., Dutta P., Sahoo M.R., Mohanty S., Kumar S., Choudhary A.K., Devi E.L., Sinyorita S. (2024). Unlocking the Potential of Arbuscular Mycorrhizal Fungi: Exploring Role in Plant Growth Promotion, Nutrient Uptake Mechanisms, Biotic Stress Alleviation, and Sustaining Agricultural Production Systems. J. Plant Growth Regul..

[303] Harbort C.J., Hashimoto M., Inoue H., Niu Y., Guan R., Rombola A.D., Kopriva S., Voges M.J.E.E.E., Sattely E.S., Garrido-Oter R. (2020). Root-Secreted Coumarins and the Microbiota Interact to Improve Iron Nutrition in *Arabidopsis*. Cell Host Microbe.

[304] Chen M.Q., Ding Z.Q., Zhou M., Shang Y.K., Li C.L., Li Q.F., Bu T.L., Tang Z.Z., Chen H. (2024). The diversity of endophytic fungi in Tartary buckwheat (*Fagopyrum tataricum*) and its correlation with flavonoids and phenotypic traits. Front. Microbiol..

[305] Han F., Xiao Y., Lee I.-S. (2020). Microbial Transformation of Prenylquercetins by *Mucor hiemalis*. Molecules.

[306] Song M.C., Kim E.J., Kim E., Rathwell K., Nam S.-J., Yoon Y.J. (2014). Microbial biosynthesis of medicinally important plant secondary metabolites. Nat. Prod. Rep..

[307] Kou X., Chai L., Yang S., He Y., Wu C.E., Liu Y., Zhou J., Xue Z., Wang Z. (2021). Physiological and metabolic analysis of winter jujube after postharvest treatment with calcium chloride and a composite film. J. Sci. Food Agric..

[308] Suzuki S., Koeduka T., Sugiyama A., Yazaki K., Umezawa T. (2014). Microbial production of plant specialized metabolites. Plant Biotechnol..

[309] Dobbelaere S., Vanderleyden J., Okon Y. (2003). Plant growth-promoting effects of diazotrophs in the rhizosphere. Crit. Rev. Plant Sci..

[310] Qadir M., Hussain A., Iqbal A., Shah F., Wu W., Cai H. (2024). Microbial Utilization to Nurture Robust Agroecosystems for Food Security. Agronomy.

[311] Najar B., Zrig A., Alsherif E.A., Selim S., Aloufi A.S., Korany S.M., Nhs M., Aldilam M., Bouqellah N.A. (2024). Synergistic Effect of Arbuscular Mycorrhizal Fungi and Germanium on the Growth, Nutritional Quality, and Health-Promoting Activities of *Spinacia oleracea* L.. Plants.

[312] Linden M., Flegler A., Feuereisen M.M., Weber F., Lipski A., Schieber A. (2023). Effects of flavonoids on membrane adaptation of food-associated bacteria. Biochim. Biophys. Acta-Biomembr..

[313] Abbaszadeh-Dahaji P., Atajan F.A., Omidvari M., Tahan V., Kariman K. (2021). Mitigation of Copper Stress in Maize (*Zea mays*) and Sunflower (*Helianthus annuus*) Plants by Copper-resistant *Pseudomonas* Strains. Curr. Microbiol..

[314] Zhou C., Ge N., Guo J., Zhu L., Ma Z., Cheng S., Wang J. (2019). *Enterobacter asburiae* Reduces Cadmium Toxicity in Maize Plants by Repressing Iron Uptake-Associated Pathways. J. Agric. Food Chem..

[315] Angelini L.G.G., Tavarini S., Ascrizzi R., Flamini G., Vannacci G., Sarrocco S. (2022). Composition and antifungal activity of the essential oils hydrodistilled from three accessions of pastinocello carrot (*Daucus carota* L. ssp. major). Ind. Crops Prod..

[316] Luciardi M.C., Amparo Blazquez M., Alberto M.R., Cartagena E., Arena M.E. (2020). Grapefruit essential oils inhibit quorum sensing of Pseudomonas aeruginosa. Food Sci. Technol. Int..

[317] Mastelic J., Politeo O., Jerkovic I., Radosevic N. (2005). Composition and antimicrobial activity of *Helichrysum italicum* essential oil and its terpene and terpenoid fractions. Chem. Nat. Compd..

[318] Chu P.H., Jenol M.A., Phang L.-Y., Syed Muhammad S.K., Abd-Aziz S. (2022). Functional Properties of Pineapple Plant Stem for Enhanced Glucose Recovery in Amino Acids Production. Energies.

[319] Das K., Prasanna R., Saxena A.K. (2017). Rhizobia: A potential biocontrol agent for soilborne fungal pathogens. Folia Microbiol..

[320] Consentino B.B., Sabatino L., Vultaggio L., Rotino G.L., La Placa G.G., D’Anna F., Leto C., Iacuzzi N., De Pasquale C. (2022). Grafting Eggplant Onto Underutilized *Solanum* Species and Biostimulatory Action of *Azospirillum brasilense* Modulate Growth, Yield, NUE and Nutritional and Functional Traits. Horticulturae.

[321] Narayanan Z., Glick B.R. (2022). Secondary Metabolites Produced by Plant Growth-Promoting Bacterial Endophytes. Microorganisms.

[322] Avci F.G., Atas B., Aksoy C.S., Kurpejovic E., Toplan G.G., Gurer C., Guillerminet M., Orelle C., Jault J.M., Akbulut B.S. (2019). Repurposing bioactive aporphine alkaloids as efflux pump inhibitors. Fitoterapia.

[323] Wang Z., Chen Z., Kowalchuk G.A., Xu Z., Fu X., Kuramae E.E. (2021). Succession of the Resident Soil Microbial Community in Response to Periodic Inoculations. Appl. Environ. Microbiol..

[324] Berg G. (2009). Plant-microbe interactions promoting plant growth and health: Perspectives for controlled use of microorganisms in agriculture. Appl. Microbiol. Biotechnol..

[325] Dong L., Li Y., Xu J., Yang J., Wei G., Shen L., Ding W., Chen S. (2019). Biofertilizers regulate the soil microbial community and enhance *Panax ginseng* yields. Chin. Med..

[326] Kumar S., Bauddh K., Barman S.C., Singh R.P. (2014). Organic Matrix Entrapped Bio-fertilizers Increase Growth, Productivity, and Yield of *Triticum aestivum* L. and Transport of NO_3_^−^, NO_2_^−^, NH_4_^+^ and PO_4_^−3^ from Soil to Plant Leaves. J. Agric. Sci. Technol..

[327] Quoc K.N., Le Vinh T., Le Thanh Q., Ngoc H.T., Do Thi X., Huu D.H., Thanh X.L.N., My T.L.T. (2024). Effects of biofertilizer supplementation, *Rhodopseudomonas* spp. on nitrogen and phosphorus uptakes, growth, and yield of sesame (*Sesamum indicum* L.) on salt-affected soil. J. Plant Nutr..

[328] Lang A.K., Jevon F.V., Vietorisz C.R., Ayres M.P., Hatala Matthes J. (2021). Fine roots and mycorrhizal fungi accelerate leaf litter decomposition in a northern hardwood forest regardless of dominant tree mycorrhizal associations. New Phytol..

[329] Ghorui M., Chowdhury S., Balu P., Burla S. (2024). Arbuscular Mycorrhizal inoculants and its regulatory landscape. Heliyon.

[330] Hezakiel H.E., Thampi M., Rebello S., Sheikhmoideen J.M. (2024). Biopesticides: A Green Approach Towards Agricultural Pests. Appl. Biochem. Biotechnol..

[331] Ragasruthi M., Balakrishnan N., Murugan M., Swarnakumari N., Harish S., Sharmila D.J.S. (2024). *Bacillus thuringiensis* (*Bt*)-based biopesticide: Navigating success, challenges, and future horizons in sustainable pest control. Sci. Total Environ..

[332] Nafis A., Elhidar N., Oubaha B., Samri S.E., Niedermeyer T., Ouhdouch Y., Hassani L., Barakate M. (2018). Screening for Non-polyenic Antifungal Produced by Actinobacteria from Moroccan Habitats: Assessment of Antimycin A19 Production by Streptomyces albidoflavus AS25. Int. J. Mol. Cell. Med..

[333] Orozco-Mosqueda M.d.C., Santoyo G., Glick B.R. (2023). Recent Advances in the Bacterial Phytohormone Modulation of Plant Growth. Plants.

[334] Shome S., Barman A., Solaiman Z.M. (2022). Rhizobium and Phosphate Solubilizing Bacteria Influence the Soil Nutrient Availability, Growth, Yield, and Quality of Soybean. Agriculture.

[335] Sogut S., Cig F. (2019). Determination of the effect of plant growth promoting bacteria on wheat (*Triticum aestivum* L.) development under salinity stress conditions. Appl. Ecol. Environ. Res..

[336] Armada E., Barea J.-M., Castillo P., Roldan A., Azcon R. (2015). Characterization and management of autochthonous bacterial strains from semiarid soils of Spain and their interactions with fermented agrowastes to improve drought tolerance in native shrub species. Appl. Soil Ecol..

[337] Emmanuel O.C., Babalola O.O. (2020). Productivity and quality of horticultural crops through co-inoculation of arbuscular mycorrhizal fungi and plant growth promoting bacteria. Microbiol. Res..

